# A method for quantitative measurement of lumbar intervertebral disc structures: an intra- and inter-rater agreement and reliability study

**DOI:** 10.1186/2045-709X-21-26

**Published:** 2013-08-16

**Authors:** Andreas Tunset, Per Kjaer, Shadi Samir Chreiteh, Tue Secher Jensen

**Affiliations:** 1 Department of Sports Science and Clinical Biomechanics, University of Southern Denmark, Campusvej 55, Odense M DK-5230, Denmark; 2 Research Department, Spine Centre of Southern Denmark, Lillebaelt Hospital, Oestre Hougvej 55, Middelfart DK-5500, Denmark; 3 DELTA, Venlighedsvej 4, Hørsholm DK-2970, Denmark

**Keywords:** Magnetic resonance imaging, Intervertebral disc, Disc herniations, Measurement, Spinal canal, Dural sac, Agreement, Reliability, Limits of agreement, Intraclass correlation coefficient

## Abstract

**Background:**

There is a shortage of agreement studies relevant for measuring changes over time in lumbar intervertebral disc structures. The objectives of this study were: 1) to develop a method for measurement of intervertebral disc height, anterior and posterior disc material and dural sac diameter using MRI, 2) to evaluate intra- and inter-rater agreement and reliability for the measurements included, and 3) to identify factors compromising agreement.

**Methods:**

Measurements were performed on MRIs from 16 people with and 16 without lumbar disc herniation, purposefully chosen to represent all possible disc contours among participants in a general population study cohort. Using the new method, MRIs were measured twice by one rater and once by a second rater. Agreement on the sagittal start- and end-slice was evaluated using weighted Kappa. Length and volume measurements were conducted on available slices between intervertebral foramens, and cross-sectional areas (CSA) were calculated from length measurements and slice thickness. Results were reported as Bland and Altman’s limits of agreement (LOA) and intraclass correlation coefficients (ICC).

**Results:**

Weighted Kappa (*K*_w_ (95% CI)) for start- and end-slice were: intra-: 0.82(0.60;0.97) & 0.71(0.43;0.93); inter-rater: 0.56(0.29;0.78) & 0.60(0.35;0.81). For length measurements, LOA ranged from [−1.0;1.0] mm to [−2.0;2.3] mm for intra-; and from [−1.1; 1.4] mm to [−2.6;2.0] mm for inter-rater. For volume measurements, LOA ranged from [−293;199] mm^3^ to [−582;382] mm^3^ for intra-, and from [−17;801] mm^3^ to [−450;713] mm^3^ for inter-rater. For CSAs, LOA ranged between [−21.3; 18.8] mm^2^ and [−31.2; 43.7] mm^2^ for intra-, and between [−10.8; 16.4] mm^2^ and [−64.6; 27.1] mm^2^ for inter-rater. In general, LOA as a proportion of mean values gradually decreased with increasing size of the measured structures. Agreement was compromised by difficulties in identifying the vertebral corners, the anterior and posterior boundaries of the intervertebral disc and the dural sac posterior boundary. With two exceptions, ICCs were above 0.81.

**Conclusions:**

Length measurements and calculated CSAs of disc morphology and dural sac diameter from MRIs showed acceptable intra- and inter-rater agreement and reliability. However, caution should be taken when measuring very small structures and defining anatomical landmarks.

## Background

In 1934, Mixter and Barr introduced the concept of lumbar disc herniations (LDH) as an explanation for radiating pain to the lower extremities [1,2]. Since then, extensive effort has been put into investigating the pathogenesis, clinical presentation, treatment and morphological changes involved in LDH [2]. LDH is generally regarded as a potential source of low back pain (LBP) and/or pain radiating to the leg, often below the knee [3]. In patients with clinical signs of nerve root compromise, about nine out of ten patients have disc-related findings on magnetic resonance imaging (MRI) [4]. On the other hand, LDH may be present without any pain or other clinical symptoms [5].

Dural sac size and intervertebral disc height have previously been found to be related to LDH, either clinically or biologically. The dural sac has a direct anatomical relationship with the intervertebral disc [6], and a direct mechanical influence is therefore possible due to an LDH taking up space in the spinal canal [7]. In addition, a correlation between a narrowed spinal canal and LBP and/or leg pain has been reported in cross-sectional studies [8-10]. Intervertebral disc height is possibly affected by LDH as material migrates posteriorly from the disc herniation. A study has shown a correlation between the classification of extended disc contour and disc height [11]. As there is evidence that disc height reduction is associated with LDHs and thus of potential clinical relevance, it was included in the current study.

Anterior disc material is similarly relevant, since it has been proposed that anterior LDHs may cause pain and symptoms [12,13]. Though this condition is rare, this imaging finding was also included in the current study, in order to be comprehensive.

Good long-term prognosis over a follow-up period of 6 months has been reported for a majority of people with LDH [14-17], and forms the current understanding of LDH among health care professionals [18,19]. In the context of clinical prognosis, it is relevant to know how LDHs change in size over time. Previous studies evaluating the change in size of LDHs over time have focused mainly on symptoms in clinical study populations [16,20-24]. Some studies have investigated the quantitative change in size of LDHs over time based on diagnostic imaging [25-29]. Three of these studies have reported the quantitative change in size over time of disc material relative to the spinal canal at multiple follow-ups [27-29], where measurements were based on a method developed by Kato et al. [27]. However, this method is described in insufficient detail to be replicated, due to the absence of definitions of anatomical boundaries.

For evaluation of disc changes over time, the ideal method is to use measurements from multiple image slices. The value of a multi-slice approach is that multiple length and area measurements can be combined into cross-sectional areas (CSA) or volumes, respectively, thereby increasing the chance of capturing changes that might otherwise be missed from single-slice methods. This multi-slice approach has been used in several studies [30-34]. It is also desirable that the method be described in sufficient detail to allow replication. Studies have provided method descriptions in varying detail [30,35-38] and in some cases, this detail is inadequate for replication.

Bland and Altman´s Limits of Agreement (LOA) is the most popular [39], and recommended statistical method for evaluation of agreement [40-44]. The standard error of measurement (SEM) is similarly regarded as a suitable parameter of agreement [45], but is, however, sensitive to variability in the population [46]. Although a recent study reported use of LOA for evaluating agreement of measurements on intervertebral disc morphology [47], it is rarely used when evaluating agreement in the measurement of intervertebral discs, LDH, or the spinal canal [48].

No method for quantitatively measuring intervertebral discs, LDH, and the dural sac was found in the literature that described in adequate detail a multi-slice technique and used LOA (Additional file 1). For a series of planned studies, we required a method to evaluate the changes in size over time of LDHs and their influence over time on dural sac size and intervertebral disc height, and their relationship with LBP. Therefore, we had need of a multi-slice technique for evaluating size of structures that was described in adequate detail and that used LOA to evaluate agreement.

The objectives of this study were:

1) to develop methods for quantitative measurement of anterior and posterior disc heights, extension of anterior and posterior lumbar disc material and dural sac diameter on MRI,

2) to evaluate the intra- and inter-rater agreement and reliability of the measurements included in these methods, and

3) to identify sources of measurement error in the measurement procedures.

## Materials and methods

### Design

The study is an intra- and inter-rater reliability study using repeated measurements of individual MRIs.

### Study population

The sample of MRIs was selected from the longitudinal cohort-study entitled ‘Backs on Funen, Denmark’, which investigated potential risk factors for LBP. The Office of Civil Registrations sampled a cohort of 40-year old Danes in 2000. All subjects were from the general population living in the county of Funen, Denmark. One out of nine people in this age group was selected (625 individuals) and invited to participate by postal mail. People were excluded if they were severely disabled, had ferromagnetic implants, suffered from claustrophobia, or were not able to communicate in Danish [49]. From this cohort, 412 participated in 2001 at baseline and were re-invited to take part in 2005. At the second measurement of the cohort in 2005, 348 participated and were re-invited to take part in 2009. At the last measurement in 2009, 293 participated. At every measurement of the cohort, all participants had a lumbar MRI and filled in a questionnaire about their LBP. Permission for the original cohort study was granted by the local ethics committee (ref. no. 20000042) and the Danish Data Protection Agency (ref. no. 2000-53-0037) [49].

Sixteen participants assessed as having a disc herniation were purposefully selected by one of the co-authors not involved in the actual measurements (PK) to represent cases with all available types of disc herniations based on previous readings of the MRIs (see below). In the upper lumbar spine, LDH was found to be almost non-existent; therefore, we chose only the three lowest levels. A list of identification numbers, levels, types of herniation, and time of examination was generated and the sample was selected to be truly representative of all types of LDH. Sixteen other participants assessed as not having a disc herniation were randomly selected to participate in the agreement analysis as controls for comparison. Only one MRI per patient was selected among the three MRIs taken at the three available time-points.

### MRI

MRI scans were performed with an open, low field 0.2 T magnetic resonance unit (Magnetom Open Viva, Siemens AG, Erlangen, Germany). The lumbar spine was scanned with participants in the supine position, using a combined body/surface coil. Sagittal T1- and T2-weighted and axial T2-weighted MRIs were performed with axial images placed in the plane of the five lower discs. The following sequences were performed at all three time-points:

• A localiser sequence of five images, 40/10/40 degrees (TR/TE/flip angle) consisting of two coronal and three sagittal images in orthogonal planes.

• Sagittal T1-weighted spin echo, 621/26 (TR/TE), 144 × 256 matrix, 300 mm. FOV, 11 slices of 4 mm. thickness, interslice gap of 0.8 mm., 2 acquisitions, 6 min. 1 sec. scan time.

• Sagittal T2-weighted turbo spin echo 4609/134 (TR/effective TE), 210 × 256 matrix, 300 mm. FOV, 11 slices of 4 mm. thickness, interslice gap of 0.8 mm., 2 acquisitions, 8 min. 42 sec. scan time.

• Axial T2-weighted turbo spin echo 6415/134 (TR/effective TE), 180 × 256 matrix, 250 mm. FOV, 3 slices of 5 mm. thickness, interslice gap of 1.0 mm., 2 acquisitions, 7 min. 49 sec. scan time. Slices were placed in the plane of the five lower discs.

To account for scoliosis and vertebral rotation, the radiographers were instructed to align the sagittal images in the best way possible in all three planes. This meant that more than one sagittal series might have been performed in cases of serious scoliosis or vertebral rotation. For the purpose of this study, only the sagittal series that had the best alignment was used for measurement.

An experienced musculoskeletal radiologist evaluated the MRI scans of the lumbar spine from all three time-points using a standardised evaluation protocol [50].

### Raters

Inter-rater agreement was tested between two raters: one of whom was a student enrolled in a Master degree in clinical biomechanics (AT) who had no prior training in the interpretation of MRIs (Rater 1); the other was an experienced back pain researcher (TSJ) with extensive experience in interpreting MRIs for research purposes (Rater 2). These raters were purposely chosen to represent an inexperienced, and an experienced, interpreter of MRI. The intra-rater agreement was tested between measures performed by Rater 1.

### Development of measurement method

Various methods for measuring the anatomical structures from MRI investigated in the current study have been reported previously [7-10,30-38,48,51-72] (Additional file 1). None of these articles described an ideal method for detecting the longitudinal change in size of LDH. A new method was therefore developed based on knowledge from the literature and the experience of the authors (AT, PK & TSJ).

Sagittal T2-weigthed MRIs were chosen for the measurements. We chose to use sagittal images because only three axial slices were available for each disc level in this study. The T2- rather than the T1-weighted sequence was chosen because of the increased contrast between the cerebrospinal fluid and the posterior part of the intervertebral disc and dural sac. Measures of length, cross-sectional area and volume were taken at the disc levels L3-L4, L4-L5 and L5-S1.

**Figure 1:**
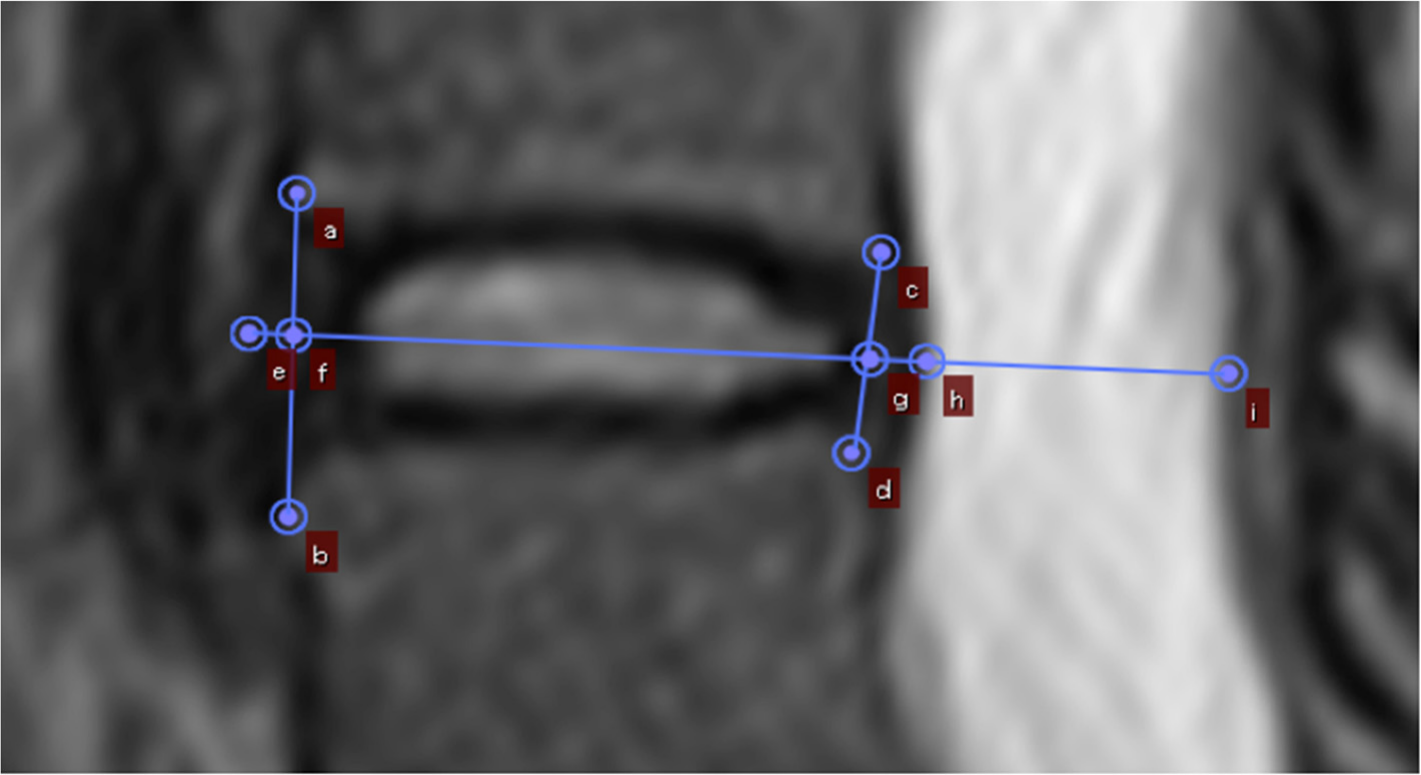
**Positioning of measured structures (a-i); (a-b) Anterior intervertebral height; (c-d) Posterior intervertebral height; (e-f) Anterior disc material; (f-g) Intervertebral disc; (g-h) Posterior disc material; (h-i) Dural sac.**

The following length measurements were defined: anterior and posterior intervertebral height (AIVH, PIVH), and the horizontal dimensions of the intervertebral disc (IVDL), anterior and posterior disc material extending beyond the corners of the vertebra (ADML, PDML) and dural sac. From these measures it was possible to calculate cross-sectional areas (CSAs): CSA of the anterior intervertebral height (CAIH), CSA of the posterior intervertebral height (CPIH), CSA of the intervertebral disc (CIVD), CSA of the anterior disc material (CADM), CSA of the posterior disc material (CPDM)s, and CSA of the dural sac (CDS). Furthermore, volume measurements were also defined for the anterior and posterior disc material that extended beyond the vertebral rim. The definitions of measurement parameters and descriptions of their mode of application are shown in Figure 1 and Table 1.

**Table 1:** Abbreviations and definitions for measurement parameters

**Measurements &calculations**	**Definitions of measurement parameters**	**Details of measurement execution**
**Length measurements**		
Anterior intervertebral height (AIVH)	Distance between anterior-superior and anterior-inferior corners at vertebrae located at relevant intervertebral disc	OsiriX ´length-tool` between most anterior point at superior corner and most anterior corner at inferior corner (Figure 1: a-b)
Posterior intervertebral height (PIVH)	Distance between posterior-superior and posterior-inferior corners at vertebrae located at relevant intervertebral disc	OsiriX ´length-tool` between most posterior point at superior corner and most posterior corner at inferior corner (Figure 1: c-d)
Intervertebral disc length (IVDL)	Distance between anterior and posterior boundaries of intervertebral disc	OsiriX ´length-tool` between midway of AIVH and midway of PIVH (Figure 1: f-g)
Anterior disc material length (ADML)	Distance between anterior and posterior boundaries of anterior herniated disc material	OsiriX ´length-tool` between most anterior located boundary of anterior disc material and midway of AIVH. Linear continuation of IVDL (Figure 1: e-f)
Posterior disc material length (PDML)	Distance between anterior and posterior boundaries of posterior herniated disc material	OsiriX ´length-tool` between midway of PIVH and most posterior located boundary of posterior disc material. Linear continuation of IVDL (Figure 1: g-h)
Antero-posterior dural sac length (ADSL)	Distance between anterior and posterior boundaries of dural sac	OsiriX ´length-tool` between most posterior located boundary of posterior disc material and most posterior located boundary of dural sac. Linear continuation of PDML (Figure 1: h-i)
**Cross**-**sectional area (CSA) calculations**		
CSA of anterior intervertebral height (CAIH)	Sum of areas estimated by the product of length measurements of anterior intervertebral height, slice thickness, and inter-slice gap distance (Figure 2a)	Calculation of CSA using all slices for AIVH length measurements. (Additional file 2: Calculating software)
CSA of posterior intervertebral height (CPIH)	Sum of areas estimated by product of length measurements of posterior intervertebral height, slice thickness, and interslice gap distance (Figure 2c)	Calculation of CSA using all slices for PIVH length measurements. (Additional file 2: Calculating software)
CSA of intervertebral disc (CIVD)	Sum of areas estimated by product of length measurements of intervertebral disc, slice thickness, and interslice gap distance (Figure 2b)	Calculation of CSA using all slices for IVDL length measurements. (Additional file 2: Calculating software)
CSA of anterior disc material (CADM)	Sum of areas estimated by product of length measurements of anterior disc material, slice thickness, and interslice gap distance	Calculation of CSA using all slices for ADML length measurements. (Additional file 2: Calculating software)
CSA of posterior disc material (CPDM)s	Sum of areas estimated by product of length measurements of posterior disc material, slice thickness, and interslice gap distance (Figure 2d)	Calculation of CSA using all slices for PDML length measurements. (Additional file 2: Calculating software)
CSA of dural sac (CDS)	Sum of areas estimated by product of length measurements of dural sac, slice thickness, and interslice gap distance	Calculation of CSA using all slices for ADSL length measurements. (Additional file 2: Calculating software)
**Volume measurements**		
Volume of anterior disc material (VADM)s	Calculated volume of anterior disc material, from tracing of sagittal areas in all slices	OsiriX ´pencil-tool` tracing area of anterior disc material anterior of AIVH at all chosen slices. Osirix ´Compute volume…` tool for volume read-out (Figure 3: a)
Volume of posterior disc material (VPDM)s	Calculated volume of posterior disc material, from tracing of sagittal areas in all slices	OsiriX´pencil-tool` tracing area of posterior disc material posterior of PIVH at all chosen slices. Osirix ´Compute volume…` tool for volume read-out (Figure 3: b)

Abbreviations used throughout the study, detailed definition of all measurement parameters, and details of measurement execution listed in sequence applied.

### Training of raters

For the training sessions, 10 participants from the final data collection period, who were judged by the radiologist to have LDH only at this time point, were randomly selected for training. Prior to the actual agreement study, each rater reviewed the 10 cases independently, after which the cases were collectively reviewed and consensus reached on the measurement procedures.

### Measurements

**Figure 2:**
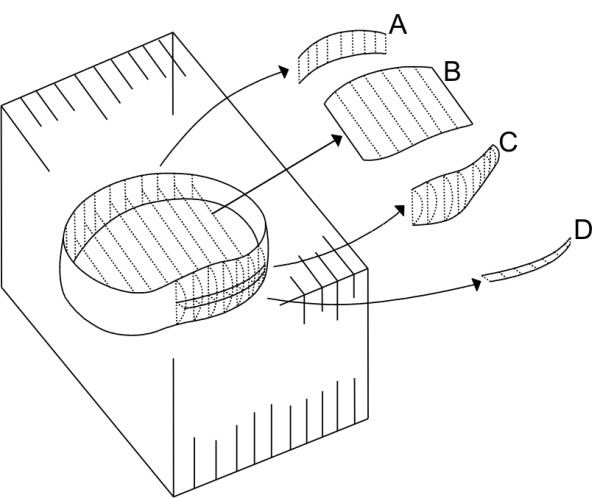
**Schematic drawing of 3D cross**-**sectional areas (CSA) and volume of disc measures from sagittal image slices. a)** CSA of anterior intervertebral height (AIVH), **b)** CSA of intervertebral disc (CIVD), **c)** Volume of posterior disc material (VPDM) and **d)** CSA of posterior disc material (CPDM).

**Figure 3:**
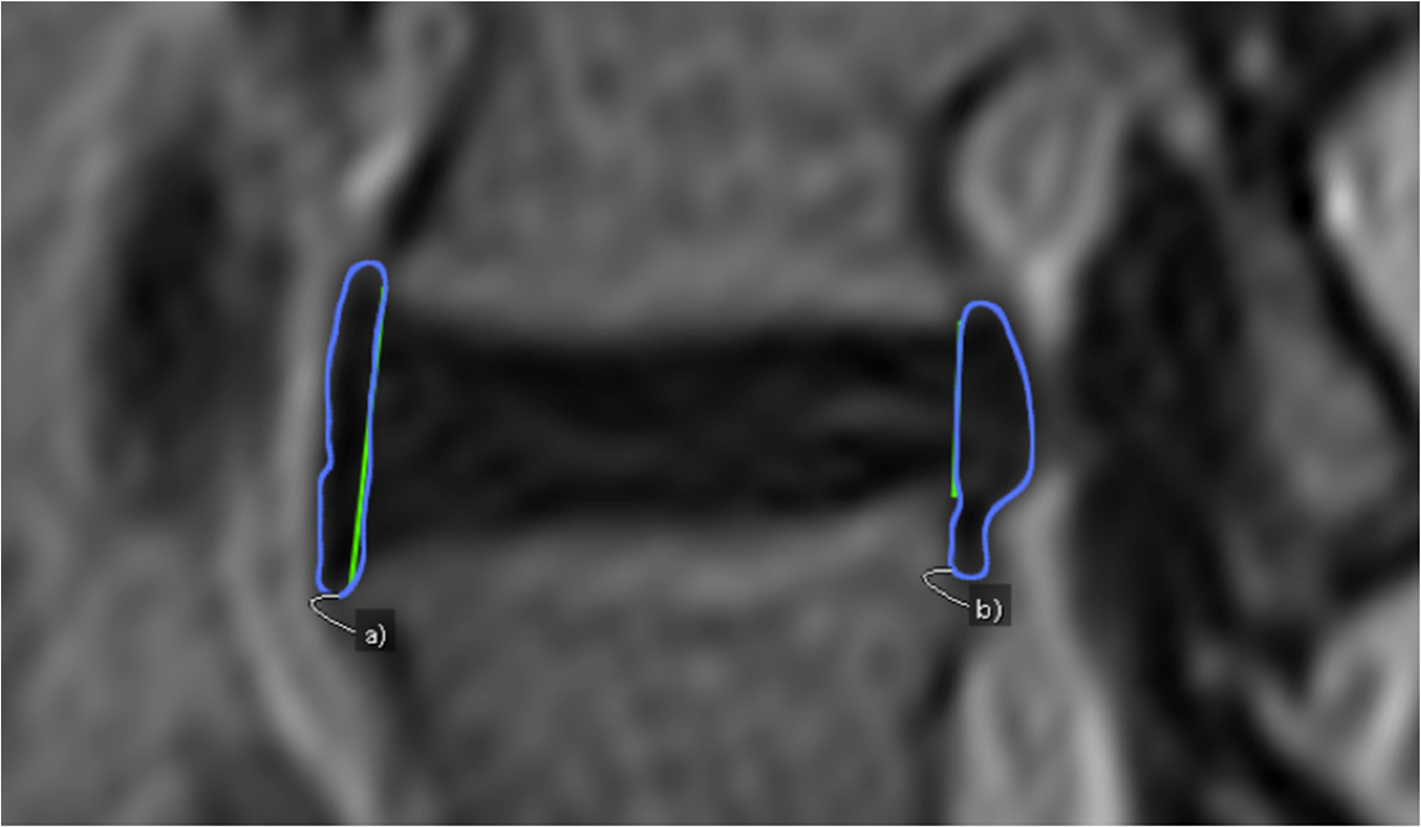
**Illustration of outlining used for volume measurements.** Outlining regions of interest in sagittal areas of **a)** anterior and **b)** posterior disc material. Volume calculated from combined areas from all slices, slice thickness, and interslice gaps. The pre-set boundary between the vertebral corners and visual boundaries completes the outlining.

All measurements were evaluated for the appropriate disco-vertebral segments on each sagittal T2-image from the first left image with a visible pedicle (start slice) to the last right image with a visible pedicle (end slice), delineating the bottom and top of an intervertebral foramina (Figure 1). All images were magnified between 1100%-1200% during measurements, showing the relevant intervertebral disc horizontally on the screen. For brightness and contrast, default settings of images were used. Length measurements were conducted using the OsiriX ´length-tool`. Length measurements taken from all included sagittal MRIs from every structure were used for calculating the CSAs of those structures (Figures 1 and 2). Volume measurements were calculated by means of OsiriX measurement software using the ‘pencil-tool’ for manually tracing regions of interest (ROIs) from all slices on each sagittal image, and the ‘Compute volume…’ tool (Figures 2 and 3).

Insertion positions on the corners of the vertebrae were defined as the most anterior point for anterior corners, and the most posterior point for posterior corners. Possible osteophytes were regarded as part of the vertebral body, as delineation of these was challenging. Insertion positions on the boundaries between structures were defined by the point showing the most contrast between structures (Figure 1). The tracing of disc material areas, used for calculating volumes, was defined as the dark visual material located anteriorly or posteriorly to the already inserted line for disc height (Figure 3). Disc material protruding inferiorly or superiorly was included until visual delineation became indistinct, because alternative ways of distinguishing outlines of disc material and its segregation from adjacent longitudinal ligaments were all more challenging. A three-dimensional illustration of the approach for measuring and calculating structures is shown in Figure 2.

To avoid potential bias due to differences of equipment and software both raters used Apple 13” MacBooks with integrated touchpads. The free open-source measurement software OsiriX (version 4.1.2) was used by both raters. This version of OsiriX is designed for scientific use [73].

Data generated from length and volume measurements were stored as comma-separated values (CSV) files, using the OsiriX ROI plugin-tool ‘export ROI’. CSV files were named with identification number, segment number, and the first and last section numbers of the MRI scan. In scans containing sections with fewer measurements of dural sac length, additional naming information was included. This naming added brackets following the initial section’s numbers containing missing dural sac identifiers. CSV files were further converted into XLSX files and converted into spread-sheets by customised software (Additional file 2) designed specifically for this study by an engineer (SSC) at the Institute of Sports Science and Clinical Biomechanics at the University of Southern Denmark, Odense, Denmark. The customised software calculated the length from the X, Y coordinates from the measurements. Calculation of CSA included the number of slices measured slice thickness, as well as the interslice gap. The CSA of the anterior intervertebral height (CAIH) and the CSA of the posterior intervertebral height (CPIH) showed the CSA in the frontal plane and the remaining CSA in the axial plane (Figure 2).

**Figure 4:**
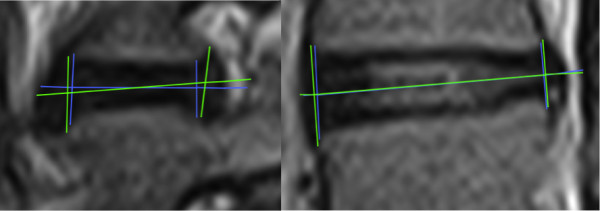
**Examples of stored measurement images**, **used in data validation.** Measurements stored as regions of interest during measurements were used for data validation. Single measurements were localised if needed and were checked against each other to ensure correct results. Images show a set of measurements with somewhat poor agreement between two measurements, and one with almost perfect agreement.

Measurement data extracted by the custom-made software and stored in Excel were checked for consistency against the original ROI files supplied by OsiriX. All calculated results were screened for obvious errors by comparing them with the ROI files (Figure 4). Errors due to any altered order of measurements were manually corrected.

### Blinding

To enhance the quality and applicability of the study, the raters were blinded in several ways [74]. Each rater was blinded to the findings of the other rater during measurements in the inter-rater analysis. In the intra-rater analysis, the rater was blinded to his own prior measurements. This was achieved by storing the data from the first measurement on a portable flash memory stick, which was stored by another project colleague. The order of participants was randomly changed between the two intra-rater measurement sessions. There was an 11-day interval between the first and second measurement sessions to lessen the likelihood of recognition of participants. All participants were anonymised for name, birth date, project ID, MRI access number, examination date, gender, and scan location.

### Data analysis

An important issue when comparing measures is whether they are performed on the same slices. Therefore, we recorded all slice numbers and compared the raters’ selections. The intra- and inter-rater agreement about the selection of the first (1, 2, 3 or 4) and last slice (6, 7, 8 or 9) for measuring sagittal images (disc parameters and dural sac), were analysed using weighted Kappa statistics and reported as weighted Kappa coefficients (*K*_w_) with 95% CI. Since our focus was on the between-rater agreement of the measurements, we only compared measures that we performed on the same slice. For volume measurements and CSA calculations, the sets of data from all subjects where the start and end slice were not the same were excluded from the analysis.

**Figure 5:**
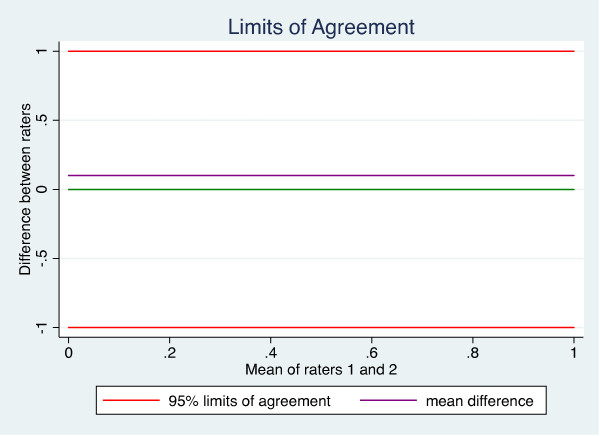
**The Bland and Altman's plot.** The y-axis shows the difference between raters’ measurements, and the x-axis shows the mean value of both raters’ measurements. The purple line shows the mean difference between measurements. Red lines show the 95% Limits of Agreement, between which 95% of all measurement differences are located.

**Figure 6:**
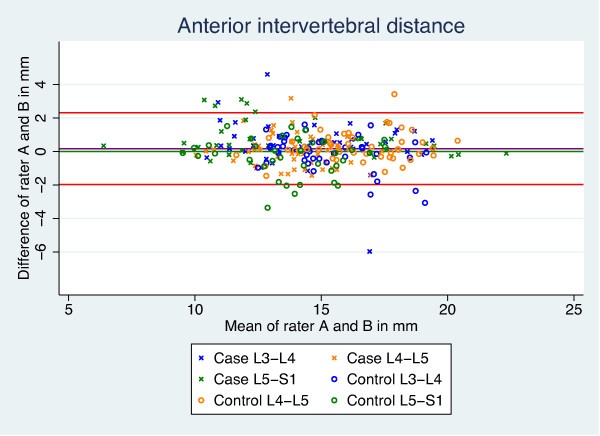
**Bland and Altman’s plot.** Example of a good result for length of anterior intervertebral distance.

**Figure 7:**
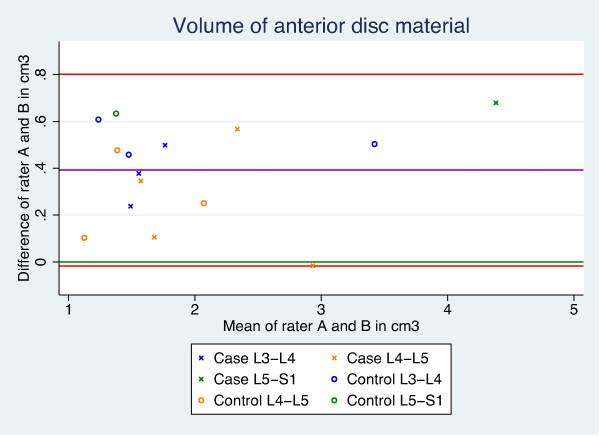
**Bland and Altman’s plot.** Example of a poor result for volume of anterior disc material.

The intra- and inter-rater agreement of the length and volume measurements, as well as the CSA calculations, were analysed using Bland & Altman’s [41] LOA. LOA is based on graphical techniques and simple calculations, and provides a plot of differences between the means of the measures, a bias shown as the mean difference, as well as the SD of the differences. This enables the calculation of 95% LOA to define ranges within which most differences between measures will lie (Figure 5). The 95% CI was reported to describe the precision of the mean difference (bias). Bias was considered present if the 95% CI did not include zero. Examples of good and poor results are given in Figures 6 and 7.

Furthermore, LOA were presented as a proportion of mean values for each structure. The proportion was calculated as follows: ((upper LOA +(−1*(lower LOA))/the mean)*100. To the best of our knowledge, no reference standard for an acceptable cut-off proportion exists. Therefore, we arbitrarily considered percentages lower than 50% as an indicator of acceptable precision.

Intra- and inter-rater reliability was evaluated with ICC type 2.1 [75]. These statistical analyses were conducted with STATA statistical software package Version 12.1 [76].

### Sample size considerations

A Kappa power calculation using the formula n=2k^2^ from Haas et al. [77] for four response option categories estimated a required sample size of 32 participants. For each participant, approximately eight measurements were made for each structure.

A post hoc estimation of the precision of the LOA was also performed based on the formula suggested by Bland and Altman [41,78] and the standard deviations from the current study. Based on this, the 95% CI for LOA was 0.21 times the standard deviation (SD) for the 257 length measurements (all < 0.26 mm), 0.69 times the SD for the 24 intra-rater CSA calculations (all < 13.2 mm^2^), and 0.88 times the SD for the 15 inter-rater volume measurements (all < 262 mm^3^). These figures indicate the sample size to be sufficient for acceptable precision of LOA for the length measures and the CSA measures but not the volume measures.

According to Bonett, an approximate sample of 15 is needed for estimating ICC with an expected coefficient of 0.9, an alpha level of 5%, a width of 0.2, and two categories [79]. The number of participants and measures exceeded that which was needed for satisfactory accuracy for evaluating reliability.

### Factors that compromise agreement

After analysis, the graphs depicting LOA were examined and outliers identified by visually distinguishing measurement differences that were far above or below the LOA on the graphs. These measurements were compared with the ROI files to identify possible reasons for ‘out of range’ measurements and reported in a narrative form. An example of comparison is given in Figure 4.

### Post hoc analysis

Due to poor inter-rater agreement on the start- and end-slices in the original analysis, a post-hoc re-analysis was undertaken. The definitions of the start- and end-slices were revised to include the requirement of visualisation of a full pedicle. This second inter-rater evaluation and weighted Kappa analysis of start- and end-slice for all structures, excluding the dural sac, were performed using the new criterion. Length and volume measurements were not repeated.

## Results

### Description of all measured parameters

In total, the lumbar MRIs from 32 participants were included in this study for evaluation of both intra- and inter-rater agreement and reliability. There were 17 females and 15 males, all aged between 40 and 49 years. Of all the measurements conducted, 10 were on segment level L3-L4, 12 on segment level L4-L5 and 10 on segment level L5-S1. Of all the available posteriorly located disc materials, 12 were classified as normal, 4 as bulged 5 as focal protrusions, 5 as broad-based protrusions, 5 as extrusions and 1 as sequestration.

### Intra-rater agreement

#### Description of measured parameters

For length measurements, 258 slices were included in the analysis for each parameter. For CSA calculations and volume measurements, 24 participants were included in the analysis for each parameter and eight participants were excluded due to differing numbers of slices. The exception was for CSA calculation for ADSL, which included 25 participants in the analysis and similarly excluded seven participants due to differing numbers of slices.

#### Start- and end-slice on measurements

Weighted Kappa for the choice of start-slice on dural sac length measurements was (*K*_*w*_ (95% CI)): 0.84 (0.65 - 0.97) and on remaining structures (*K*_w_ (95% CI)): 0.82 (0.60 - 0.97)). Weighted Kappa for the end-slice on dural sac length measurements was (*K*_*w*_ (95% CI): 0.87 (0.71 - 0.97) and on all remaining structures was (*K*_*w*_ (95% CI): 0.71 (0.43 - 0.93)). Cross tabulations are available in Additional file 3.

#### Measurements of length

The mean difference of all length measurements ranged between −0.1 mm and 0.2 mm, with 95% CI ranging between −0.2 mm and 0.3 mm. LOA ranged between [−1.0; 1.0] mm and [−2.0; 2.3] mm, and between 6.8% and 62.9% of mean values (Table 2 and Additional file 4).

**Table 2:** **Intra**-**rater measures agreement results**

**Measurement**	**n (slices)**	**Mean (mm)**	**Standard deviation (mm)**	**Mean difference (bias) [95% CI] (mm)**	**95% LOA (mm)**	**LOA as proportion of mean values (%)**
Length - AIVH	258	14.7	1.1	0.2 [0.0; 0.3]	−2.0; 2.3	29.3
Length - PIVH	258	9.5	0.8	0.1 [0.0; 0.2]	−1.5; 1.6	32.6
Length - IVDL	258	31.1	0.5	−0.1 [−0.1; 0.0]	−1.1; 1.0	6.8
Length - ADML	258	3.5	0.6	0 [−0.1; 0.1]	−1.1; 1,1	62.9
Length - PDML	258	3.6	0.5	0 [−0.1; 0.1]	−1.0; 1.0	55.6
Length - ADSL	227	8.5	0.8	−0.1 [−0.2; 0.0]	−1.6; 1.4	35.3
	**n (participants)**	**(mm**^**2**^)	(**mm**^**2**^)	(**mm**^**2**^)	(**mm**^**2**^)	(%)
Area - CAIH	24	512.6	19.1	6.2 [−1.8; 14.3]	−31.2; 43.7	14.6
Area - CPIH	24	327.6	11.7	4.5 [−0.4; 9.4]	−18.4; 27.4	14.0
Area - CIVD	24	1101.3	10.2	−1.2 [−5.5; 3.1]	−21.3; 18.8	3.6
Area - CADM	24	118.8	10.2	1 [−3.3; 5.3]	−19.0; 21.1	33.8
Area - CPDM	24	121.8	12.5	−1.6 [−6.9; 3.7]	−26.0; 22.8	40.1
Area - CDS	25	267.9	18.6	−3.8 [−11.5; 3.9]	−40.3; 32.7	27.2
		(**mm**^**3**^)	(**mm**^**3**^)	(**mm**^**3**^)	(**mm**^**3**^)	(%)
Volume - VADM	24	2136.7	246	−100 [−204; 4]	−582; 382	45.1
Volume - VPDM	24	1314.8	126	−47 [−100; 6]	−293; 199	37.4

Number of slices measured for length, and participants measured for cross-sectional area and volume measurements, overall mean values, standard deviation, mean difference between measurements with 95% confidence intervals (CI), 95% limits of agreement (LOA), and LOA as a proportion of mean values. Due to absence of dural sac at certain otherwise measured slices, a lower number of slices were measured. Participants with unequal start- and end-slices were excluded from the analyses, leading to varying numbers of included participants.

#### Estimation of cross-sectional area

The mean difference of all CSA calculations ranged between −3.8 mm^2^ and 6.2 mm^2^, with 95% CI ranging between −11.5 mm^2^ and 14.3 mm^2^. LOA ranged between [−21.3; 18.8] mm^2^ and [−31.2; 43.7] mm^2^, and between 3.6% and 40.1% of mean values (Table 2 and Additional file 4).

#### Measurements of volume

Mean differences for both volume measurements were −100 mm^3^ and −47 mm^3^, with 95% CI ranging between −204 mm^3^ and 6 mm^3^. LOA ranged between [293; 199] mm^3^ and [−582; 382] mm^3^, and between 37.3% and 45.1% of mean values (Table 2 and Additional file 4).

### Intra-rater reliability

ICCs ranged from 0.90 (95% CI 0.88-0.92) to 0.99 (0.99-1.00) for length measurements and from 0.95 (0.89-0.98) to 1.00 (1.00-1.00) for CSAs. ICCs for measurement of volume were 0.95 (0.88-0.98) for anterior disc material and 0.95 (0.89-0.98) for posterior disc material (Table 3).

**Table 3:** **Intra**-**rater measures reliability results**

**Measurement**	**n** (**slices**)	**ICC** [**95**% **CI**]
Length - AIVH	258	0.91 [0.88, 0.93]
Length - PIVH	258	0.90 [0.88, 0.92]
Length - IVDL	258	0.99 [0.99, 1.00]
Length - ADML	258	0.95 [0.94, 0.96]
Length - PDML	258	0.94 [0.92, 0.95]
Length - ADSL	227	0.98 [0.98, 0.99]
	**n** (**participants**)	
Area – CAIH	24	0.99 [0.98, 1.00]
Area – CPIH	24	0.98 [0.96, 0.99]
Area - CIVD	24	1.00 [1.00, 1.00]
Area - CADM	24	0.97 [0.94, 0.99]
Area - CPDM	24	0.95 [0.89, 0.98]
Area - CDS	25	0.97 [0.93, 0.99]
Volume - VADM	24	0.95 [0.89, 0.98]
Volume - VPDM	24	0.95 [0.88, 0.98]

Number of slices measured for length and participants measured for cross-sectional area and volume measurements, intraclass correlation coefficient (ICC), and accompanying 95% confidence intervals (CI). Due to absence of dural sac at certain otherwise measured slices, a lower number of slices were measured. Participants with unequal start- and end-slices were excluded from the analyses, leading to varying numbers of included participants.

### Inter-rater agreement

#### Description of measured parameters

For length measurements, 257 slices were included in the analysis for each parameter. For CSA calculations and volume measurements, 15 participants were included in the analysis for each parameter and 17 participants were excluded due to differing numbers of slices. The exception was the CSA calculation for ADSL, which included eight participants in the analysis and excluded 24 participants due to differing numbers of slices.

#### Start- and end-slice for measurements

Weighted Kappa for the choice of start-slice on dural sac length measurements was (*K*_*w*_ (95% CI): 0.22 (0.08 - 0.42) and on remaining structures was (*K*_*w*_ (95% CI): 0.35 (0.17 - 0.56)). Weighted Kappa for the choice of end-slice on dural sac length measurements was (*K*_*w*_ (95% CI): 0.22 (0.05 - 0.43) and on all remaining structures (*K*_*w*_ (95% CI): 0.37 (0.08 - 0.66)). *Post hoc* analysis for start- and end-slice on all structures except dural sac showed weighted Kappa for start- (*K*_*w*_ (95% CI): 0.56 (0.29 - 0.78)) and for end-slice (*K*_*w*_ (95% CI): 0.60 (0.35 - 0.81)). Cross tabulations are available in Additional file 3.

#### Measurements of length

The mean difference of all length measurements ranged between −0.7 mm and 0.3 mm, with 95% CI ranging between −0.8 mm and 0.4 mm. LOA ranged between [−1.1; 1.4] mm and [−2.6; 2.0] mm, and between 9.7% and 105.9% of mean values (Table 4 and Additional file 4).

**Table 4:** **Inter**-**rater measures agreement results**

**Measurement**	**n** (**slices**)	**Mean** (**mm**)	**Standard deviation** (**mm**)	**Mean difference** (**bias**) [**95**% **CI**] (**mm**)	**95% Limits of agreement** (**LOA**) (**mm**)	**LOA as proportion of mean values (%)**
Length - AIVH	257	14.9	1.2	−0.5 [−0.7; –0.4]	−2.8; 1.8	30.9
Length - PIVH	257	9.6	1.2	−0.3 [−0.4; –0.2]	−2.6; 2.0	47.9
Length - IVDL	257	31.2	0.8	−0.7 [−0.8; –0.6]	−2.2; 0.8	9.7
Length - ADML	257	3.4	0.6	0.1 [0.0; 0.2]	−1.1; 1.4	73.6
Length - PDML	257	3.4	0.9	0.3 [−0.2; 0.4]	−1.5; 2.1	105.9
Length - ADSL	229	8	1.1	0.2 [0.0; 0.3]	−2.0; 2.3	53.8
	**n** (**participants**)	(**mm2**)	(**mm**^**2**^)	(**mm**^**2**^)	(**mm**^**2**^)	(%)
Area - CAIH	15	568.2	23.4	−18.7 [−31.7; –5.7]	−64.6; 27.1	16.2
Area - CPIH	15	362.8	17.3	−13.3 [−22.9; –3.7]	−47.3; 20.7	18.7
Area - CIVD	15	1190.4	13.7	−19.5 [−27.1; –11.9]	−46.4; 7.4	4.5
Area - CADM	15	126.2	7	2.8 [−1.1; 6.7]	−10.8; 16.4	21.6
Area - CPDM	15	121.7	15	6.4 [−2.0; 14.7]	−23.1; 35.8	48.4
Area - CDS	8	286	17.8	4.8 [−10.1; 19.7]	−30.1; 39.8	24.4
		(**mm**^**3**^)	(**mm**^**3**^)	(**mm**^**3**^)	(**mm**^**3**^)	(%)
Volume - VADM	15	1830.3	209	392 [277; 508]	−17; 801	44.7
Volume - VPDM	15	1117.6	297	131 [−33; 296]	−450; 713	104.1

Number of slices measured for length and participants measured for cross-sectional area and volume measurements, overall mean values, standard deviation, mean difference between measurements (bias) with 95% confidence intervals (CI), 95% limits of agreement (LOA), and LOA as a proportion of mean values. Due to absence of dural sac at certain otherwise measured slices, a lower number of slices were measured. Participants with unequal start- and end-slices were excluded from the analyses, leading to varying numbers of included participants.

#### Estimation of cross-sectional area

The mean difference for all CSA calculations ranged between −19.5 mm^2^ and 6.4 mm^2^, with 95% CI ranging between −31.7 mm^2^ and 19.7 mm^2^. LOA ranged between [−10.8; 16.4] mm^2^ and [−64.6; 27.1] mm^2^, and between 4.5% and 48.4% of mean values (Table 4 and Additional file 4).

#### Measurements of volume

Mean differences were 131 mm^3^ and 392 mm^3^, with 95% CI ranging between −33 mm^3^ and 508 mm^3^. LOA ranged between [−17; 801] mm^3^ and [−450; 713] mm^3^, and between 44.7% and 104.1% of mean values (Table 4 and Additional file 4).

### Inter-rater reliability

ICCs ranged from 0.73 (0.69-0.79) to 0.98 (0.90-0.99) for length measurements and from 0.88 (0.69-0.96) to 0.99 (0.97-1.00) for CSAs. ICCs for measurement of volume were 0.57 (0.13-0.83) for anterior disc material and 0.90 (0.00-0.98) for posterior disc material (Table 5).

**Table 5:** **Inter**-**rater measures reliability results**

**Measurement**	**n** (**slices**)	**ICC** [**95**% **CI**]
Length - AIVH	257	0.88 [0.82 – 0.92]
Length - PIVH	257	0.81 [0.76 – 0.85]
Length - IVDL	257	0.98 [0.90 – 0.99]
Length - ADML	257	0.93 [0.91 – 0.95]
Length - PDML	257	0.73 [0.64 – 0.79]
Length - ADSL	229	0.96 [0.95 – 0.97]
	**n (participants)**	
Area - CAIH	15	0.96 [0.81 – 0.99]
Area - CPIH	15	0.93 [0.68 – 0.98]
Area - CIVD	15	0.99 [0.78 – 1.00]
Area - CADM	15	0.99 [0.97 – 1.00]
Area - CPDM	15	0.88 [0.69 – 0.96]
Area - CDS	8	0.95 [0.79 – 0.99]
Volume - VADM	15	0.90 [0.00 – 0.98]
Volume - VPDM	15	0.57 [0.13 – 0.83]

Number of slices measured for length and participants measured for cross-sectional area and volume measurements, intraclass correlation coefficient (ICC), and accompanying 95% confidence intervals (CI). Due to absence of dural sac at certain otherwise measured slices, a lower number of slices were measured. Participants with unequal start- and end-slices were excluded from the analyses, leading to varying numbers of included participants.

### Bias estimates

The 95% CI for mean differences suggested no statistically significant bias for intra-rater measures, and suggested a possible significant bias in a negative direction for seven out of 14 inter-rater parameters.

### Factors that compromise agreement

A total of 27 outliers consisting of single intra-rater measurements and 20 outliers consisting of single inter-rater measurements were seen from the LOA plots. Three reasons were identified:

1) A different interpretation of vertebral corners at both the anterior and posterior locations, as well as superior and inferior locations was the reason for seven AIVH and PIVH outliers, nine IVDL outliers, one ADML outlier, and three PDML outliers. This may have been the reason for the IVDL and PDML outliers due to their dependence on AIVH and PIVH measurements.

2) Inconsistent distinction between structural boundaries due to lack of contrast was identified as inherent in three separate causes for outliers. The first was that five outliers were caused by a different interpretation of the anterior boundary of ADML. The second was that six outliers were caused by a different interpretation of the boundary between PDML and ADSL. The third was that fifteen outliers were caused by a different interpretation of the posterior boundary of ADSL.

3) A single outlier for each of IVDL, ADML, PDML and ADSL was identified as an error in measurement execution. These errors were included in the CSAs and therefore influenced their results.

## Discussion

This study reports a new method for measuring lumbar disc-related structures for use in research and in clinical practice. Intra-rater reliability in selecting start- and end-slice was substantial and inter-rater reliability changed from poor to moderate after revision of the method [80]. The Bland and Altman’s LOA showed very little bias (mean difference) and a small range for all intra-rater measurements and calculations. Reliability was high with most ICCs > 0.90. For inter-rater measurements and calculations the Bland and Altman’s LOA showed slightly higher bias and slightly higher ranges, with the exception of volume measurements, which had considerably larger bias and ranges. Reliability was slightly lower but most ICCs were > 0.73. The uncertainty around volume measures was considerable. In general, LOA as a percentage of the mean values gradually decreased with increased size of the measured structures.

The results indicate that when measuring very small structures (e.g. ADML and PDML) on MRI, the changes over time have to be relatively large in order to detect changes. Combining length measures into volume measures reduces the LOA as a proportion of the mean. The measurement of volume by manual tracing seems to be dependent on the observer and the VPDM seems to be particularly problematic to agree upon.

The intra-rater measurements and calculations showed better agreement than inter-rater measurements, although the differences were not large. This indicates a good consensus regarding the anatomical delineation between length measurements by the same rater, but also acceptable consensus between the two raters. The same does not apply with volume measurements, where the inter-rater agreement was not acceptable. It seems the cumulative error in the marking of multiple anatomical structures was not accurate enough between multiple raters, resulting in differences that were unacceptably high. The same applies for start- and end-slice, where it seems agreement between raters is poor unless sufficient consensus on measurements is made beforehand. This appears to be due to difficulty in determining the slice delineating the boundary of the foramina, when using the criterion of visualisation of a fully visible pedicle, a criterion previously described in the literature [81].

Outliers found during the validation of the results could generally be traced to two main reasons: one being inexact positioning of vertebral corners; the other being difficulties in distinguishing between the anterior or posterior boundaries between structures. As for positioning of vertebral corners, a possible interfering factor could be the presence of osteophytes, by their modifying the visual appearance of the vertebra. For future use of this method, specification in advance of measurements, and persistent implementation of detailed definitions for aforementioned positionings, should be conducted by all raters. We were not able to find articles that definitively discussed any of these factors regarding similar problems with positioning or boundary distinction. Videman et al. [82] previously used a more thorough method for defining ‘theoretical’ vertebral corners. However, such an approach is likely to be more complicated and time-consuming.

A similar method of measuring the spinal canal was performed by Dora et al. [8]. They used sagittal MRIs and ICC and reported good inter-rater reliability (ICC>0.95). Other studies have used similar methods for measuring the spinal canal or the dural sac, but have not documented any kind of reproducibility [9,28,63,68,69]. A similar method is also used for measuring disc herniations and the spinal canal in some studies [27-29], but the method is described inadequately, and there is no reporting of analysis of agreement or reliability. One study performed similar quantitative measurements of similar structures on MRIs using LOA for determining agreement [48]. In this study, one finding on intervertebral disc length is comparable with the current study and indicates similar LOA. That study sample consisted of children and therefore their population was not directly comparable with ours. A study that compared results of MRIs in different positions showed anteriorly and posteriorly herniated disc material length measurements with almost exactly the same values [83]. A direct comparison with other studies is difficult, as this is the first study, to our knowledge, with the current statistical approach and such a detailed description of the method.

Agreement, together with reliability, is generally embedded in the expression reproducibility. In the literature, agreement and reliability are often used interchangeably, although their foci are different. Agreement focuses on measurement error when the focus is change in health status over time, while reliability is concerned with measurement error plus the variability between study objects and the focus is distinction between persons [45]. deVet et al. recommend reporting agreement parameters such as LOA, and further, when reporting reliability using ICC, they should be reported together with error estimates such as SEM [45]. This study uses both agreement and reliability, but the clear distinction between their use and meaning has been preserved.

Our review of the available literature (Additional file 1) showed a common pattern in methodological limitations through the use of inappropriate methods for longitudinal measurements, inadequate descriptions of methods, as well as unsatisfying statistical analyses of agreement. Out of 34 studies, only 17 reported reproducibility, and only one of these studies [48] used an appropriate statistical method – in that case, LOA. Eight of the remaining studies [8,33,34,38,48,52,55,57] used ICC, which is a measure of reliability, not agreement [45]. Furthermore, only one out of these eight studies reported an error estimate [55].

We interpret our results as indicating that the measurement method used in this study is suitable for further use, with the exception of volume measurements. The method also makes it possible to validate data regarding errors made during measurements and those made during calculations, as well as indications for how to correct relevant errors in advance of the analysis. This data validation method may also be used for localising the reasons for outliers. As seen in the post-hoc analysis, a focus on consensus between raters is important for obtaining agreement about start- and end-slices. Our study is likely to be useful for future research because the method is appropriate for longitudinal measurements it contains a full and detailed description of the method and includes adequately conducted agreement and reliability analyses. In future studies and in clinical practice, this method can be used to detect changes larger than the LOA in disc morphology over time in individuals and between groups of patients. However, the size of the measure of interest has to be considered, since the relative precision increases with the size of the measurement (LOA as a percentage of the mean, Tables 2 and 4). In our research group, this method will form the basis for a series of research projects with the aims of investigating the changes in disc morphology over time and their association with clinical outcomes.

There could be a number of reasons for the observed poor agreement of inter-rater volume measurements. A possible explanation is a lack of certainty when manually tracing the anterior and posterior herniated disc material – an issue reported in earlier studies addressing volume measurements using MRIs [84,85]. Another explanation is a possible difficulty in separating herniated disc material from the longitudinal ligament, as these structures appear with almost the same signal intensity on MRI.

One limitation of this study may be the low resolution of the MRIs and the high magnification levels used. With a 144×256 matrix, 300 mm field of view and 4 mm slice thickness [49], the DICOM reader software digitally reconstructed the high detail of anatomical structures visible on the MRIs. This, in addition to the high magnification levels, increases the measurement precision but may reduce the accuracy of the image’s representativeness of the actual anatomy. Any length measurement below the size of one voxel (1.2(height) × 1.4(width) × 4.0(depth) mm) could therefore be considered relatively inaccurate. As for the length measurements of the anterior and posterior herniated disc material, there is a possibility that most of the anterior or posterior position is above or below the measured level, leading to possible underestimation of disc material sizes. Furthermore, as this study is not a test-retest study, it does not take into account the measurement errors that would be associated with repositioning patients, diurnal variations and the effect of activities within its estimates of intra- and inter-rater reliability.

The original study cohort was representative of the general population but the selection of a sample of cases and controls for the current study may affect the generalisability of the results. The reported means of measurements will not reflect those of the original cohort since only 22-25% in it had LDH. Although the prevalence of LDH, especially the more severe types, is likely to be higher in a clinical population, we believe that the measurement method will work in clinical populations. Our aim was to establish reproducibility and reliability, not to report prevalence or reference values for either a general or a clinical population.

It is possible that the ICCs and weighted Kappa values are inflated in this study, due to the large variability in the measures when purposefully selecting a sample representative of all types of LDH and of controls without LDH. The results may also be inflated by excluding a number of the more lateral MRI slices, when there was disagreement on start- and end-slice. The reason for this is that the LOA were relatively smaller for the larger structures. Another factor that may have increased the reproducibility and reliability is that only two raters were performing the measurements. However, when comparing ICCs in our study with those in other studies using the same measure of reliability, the results were very similar [8,34,38].

In this study, we have performed several statistical analyses with an alpha level of 5% which by definition increases the risk of at least one chance finding in every twenty tests. However, the trends for the LOA and the ICCs are all in the same direction for the included measures. The variability in lumbar levels, LDH and normal discs in the study sample could lead to a suspicion that the LOA would be different for certain subgroups. However, in the Bland and Altman’s LOA plots (Additional file 4), colours indicate the different levels as well as cases and controls. And when looking carefully at these, there are no obvious differences.

The strengths of this study are the high number of single length measurements, the carefully planned execution, the extensive review of the available literature as well as the well-described method. The high number of length measurements is also the basis for the CSAs. This study also followed a structured protocol from the beginning and adhered throughout to guidelines for studies of agreement [44,74]. Finally a comprehensive description of the method is available, as is the freeware measurement software [73]. This method also only takes 5 to 20 minutes per MRI to measure and interpret, depending on equipment, software preparation, and experience. In a clinical setting, a selection of relevant parameters such as CPDM, CPIH, and CDS may reduce the time consumption considerably.

## Conclusion

This new method of quantifying length measurements of disc morphology and dural sac diameter from MRIs showed good intra- and inter-rater agreement as well as reliability. Quantitative volume measurements showed unacceptable agreement and reliability. However, caution should be taken when selecting start- and end-slice, measuring very small structures, and when defining anatomical landmarks. This method for quantitative measurement of lumbar intervertebral discs and related structures is suitable for testing in broader contexts, including in more diverse clinical samples, and in quantitative research that involves serial measurement of anatomical structures over multiple follow-up time periods.

## Abbreviations

ADML: Anterior disc material length; ADSL: Antero-posterior dural sac length; AIVH: Anterior intervertebral height; AT: Andreas Tunset; BSc: Bachelor of Science; CADM: Cross-sectional area of anterior disc material; CAIH: Cross-sectional area of anterior intervertebral height; CDS: Cross-sectional area of dural sac; CI: Confidence interval; CIVD: Cross-sectional area of intervertebral disc; CPDM: Cross-sectional area of posterior disc material; CPIH: Cross-sectional area of posterior intervertebral height; CSA: Cross-sectional area; CSV: Comma separated values; DICOM: Digital imaging and communities in medicine; ICC: Intra-class correlation coefficient; ID: Identification; IVDL: Intervertebral disc length; Kw: Weighted Kappa; LBP: Low back pain; LDH: Lumbar Disc Herniation; LOA: Limits of agreement; MRI: Magnetic resonance imaging; MSc: Master of Science; PDML: Posterior disc material length; PhD: Doctor of Philosophy; PIVH: Posterior intervertebral height; PK: Per Kjaer; ROI: Region of interest; SSC: Shadi Samir Chreiteh; T: Tesla; TSJ: Tue Secher Jensen; VADM: Volume of anterior disc material; VPDM: Volume of posterior disc material.

## Competing interests

There are no competing interests among authors.

## Authors’ contributions

AT, PK and TSJ developed the concept and design and administered the study, developed the method used in the study, performed the analysis and drafted the manuscript. AT and TSJ conducted all intra- and inter-rater measurements. SSC developed the software for calculating the data. AT drafted the manuscript. PK and TSJ reviewed the manuscript several times. All authors approved the manuscript in its final form.

## Authors’ information

An additional list of each author’s qualifications and affiliations is available at the start of the article. This study is part of the undergraduate research education of a Master program in Clinical Biomechanics being undertaken by AT.

## Supplementary Material

Additional file 1 Literature review. Click here for file

Additional file 2 Description of calculating software (computer program available from the authors on request). Click here for file

Additional file 3 Cross tabulations for start- and end-slices. Click here for file

Additional file 4 Graphs of limits of agreement. Click here for file
