# Supplementary material for: A method for quantitative measurement of lumbar intervertebral disc structures: an intra- and inter-rater agreement and reliability study
Source: Chiropr Man Therap. 2013 Aug 16;21:26. doi: 10.1186/2045-709X-21-26 (PMC3751877; doi:10.1186/2045-709X-21-26)
Supplement: Additional file 1 — Literature review. [file 2045-709X-21-26-S1.pdf]

Additional file 1 – Literature review

Table 1 – Data extraction from studies included in the literature review including first author and year of publication, study design and sample of study, sample size and gender distribution, range in age of participants, imaging modality used, structures measured (variables), measurement software, main findings, and details of the reproducibility reported.

Author and year	Study design and study sample	Sample size (males + females)	Age range	MRI/CT	MRI/CT variables	Measurement software	Main findings	Reproducibility of method
Grenier 1987 [1]	Descriptive study on healthy subjects, as well as patients with degenerative changes	13 healthy subjects<br><br>30 patients	23-80	MRI & CT	Vertebral facets, cartilage, ligamentum flavum, bone marrow and spinal canal	Not reported	MR-images in the sagittal plane were useful in demonstrating hypertrophy of the ligamentum flavum or the vertebral facets, in grading the degree of foraminal stenosis, and in measuring the sagittal diameter of the spinal cord. MR images in the axial plane facilitated detailed analysis of the facet joint and more accurate measurements of the thickness of the ligamentum flavum and spinal canal diameter.	MR compared with CT in 12 patients.<br><br>Specific results for reproducibility not reported
Hamanishi 1994 [2]	Descriptive study on patients with low back pain, radicular symptoms, intermittent claudication or non-lumbar lesions	222	13-87<br>Mean 48.3	MRI	Lumbar dural sac	Computerized digitizer: Wacom WT-5000LP (Not specific software reported)	Cross-sectional areas obtained with simplified geometric formulas was highly correlated with that calculated with the digitizer, indicating that this simple method can be used with MRI in outpatient clinics for the rapid determination of the most stenotic portion of the dural tube.	Intraclass correlation coefficient<br><br>Intra-rater: 0.96<br>Inter-rater: 0.92
Carragee 1997 [3]	Observational study on patients with a primary diagnosis of sciatica	186<br>(108-78)	20-68<br>Mean 42.8	MRI	Lumbar intervertebral discs & spinal canals	General Electric (Milwaukee, WI)	Quantitative measurements by magnetic resonance imaging of disc and canal morphology of 188 patients with sciatica indicate a wide range of herniation and canal sizes, with significant differences between men and women.	Intra-rater and inter-rater errors in measurements were determined to be less than 3%.<br><br>More specific documentation of reproducibility not reported.
Malko 1999 [4]	Longitudinal descriptive MRI-study on subjects from the general population	5<br>(4+1)	27-52	MRI	Lumbar intervertebral discs	SPARC-20 Workstation (Sun Microsystems, Mountainview, CA)	Load-induced changes in disc volume can be detected and measured using magnetic resonance imaging.	Two-tailed t-test.<br>Inter-rater: (P = 0.3-0.9)<br>No significant difference between raters.
Chung 2000 [5]	Descriptive comparative study on healthy young subjects	20<br>(9+11)	22-30<br>Mean 26	MRI	Lumbar intervertebral discs & spinal canals	Picture Archiving Communication System (PACS, Loral, USA).	Extension or rotation increased thickness of ligamentum flavum. Posterior margin of intervertebral disc approximated to facet joint without any change in shape and size of the disc. Decrease in the size of the spinal canal and dural sac in extension or rotation postures	Not reported
Holodny 2000 [6]	Cross-sectional study on patients with LDH	44<br>(25+19)	22-71<br>Mean 44	MRI	LDH	Not reported	LDH causes a loss of disc height, but a significant decrease.	Between axial and sagittal measurements.<br>Correlation coefficient = 0.85
Pneumaticos 2000 [7]	Descriptive study on patients with leg pain secondary to LDH	66<br>(29+37)	18-66<br>Mean 43	MRI	LDH & spinal canals	Brain-image software (National Institute of Health Behavioural Neurogenetics	A herniated disc with an AP diameter of approximately 3 mm was over 95% sensitive and 95% specific. However, if the AP diameters of herniated discs in symptomatic patients were	Not reported

Author and year	Study design and study sample	Sample size (males + females)	Age range	MRI/CT	MRI/CT variables	Measurement software	Main findings	Reproducibility of method
						and Neuroimaging Research Centre)	compared with similar measurements in asymptomatic controls, the most sensitive and specific threshold value was 6.8 mm.	
Cooley<br>2001 [8]	Descriptive study on MRI findings.<br>Patient population with history of low back pain.	122<br>(610 lumbar intervertebral discs)	16-83<br>Mean (46)	MRI	LDH	Generic CADD 6.0 software	Different disc types demonstrated distinct size averages.	Intraclass correlation coefficient.<br>Inter-rater agreement (0.78-0.84)<br>Intra-rater agreement (0.40-0.49)
Dora<br>2002 [9]	Cross-sectional descriptive study on patients with LDH selected for discectomy, and asymptomatic subjects	75<br>(54+21)	20-50<br>Mean 35	MRI	Lumbar spinal canals	SIGNA, General Electric Medical Systems, Milwaukee, Wisconsin	In symptomatic patients, spinal canal dimensions are significantly smaller than those in asymptomatic individuals.	Pearson correlation and intraclass correlation coefficient.<br><br>Intra-rater: 0.98-0.99<br>Inter-rater: 0.95-0.99
Haig<br>2002 [10]	Descriptive study on subjects referred for electro-diagnostic testing of the low back and lower limb	44<br>(22+22)	Mean 48.5	MRI	Lumbar spinal canals	Signa, Milwaukee, WI: General Electric Medical System	The radiologist's overall impression is more accurate than axial image measurements in predicting paraspinal denervation. There is more denervation with increasing age in this symptomatic population.	Not reported
Speciale<br>2002 [11]	Descriptive study on patients with lumbar spinal stenosis	15<br>(9+6)	Mean 71.5	MRI	Lumbar spinal canals (Central, lateral, and foraminal stenosis)	Vidar Systems Corporation	Findings indicate only a fair level of agreement of predicting degree of spinal stenosis among all observers. However, the ability of the various readers to predict the degree of central stenosis was high.	Linear regression model<br><br>Only reported significant inter-rater specificity (P=0.001)
Malko<br>2002 [12]	Longitudinal descriptive MRI-study on subjects from the general population	5<br>(1+4)	21-32	MRI	Lumbar intervertebral discs	SPARC-20 Workstation (Sun Microsystems, Mountainview, CA)	The disc gains 10.6% of its volume during an 8-h overnight bed rest period. 2) The disc volume decreases after rising and walking. 3) After 8 h (using our particular walking/scanning protocol), the disc volume does not decrease to the volume measured at the end of the previous day.	Relative volume errors, based on standard deviation from multiple measurements.<br>Approximately 4%
Jeffrey<br>2003 [13]	Cross-sectional descriptive study on children from the general population	161<br>(	14-16<br>All born in 1988-1989	MRI	Lumbar spinal canals	Numaris software & Scion Image	Size of the lumbar vertebral canal was reduced by low birth weight, with maternal smoking as an added adverse factor.	Only reported no significant differences, at 20 subjects.
Carlisle<br>2005 [14]	Cross-sectional descriptive study on patients with leg pain, positive nerve root tension and/or muscle weakness	88<br>(52-36)	20-72<br>Mean 37.5	MRI	Lumbar dural sacs & LDH	Not reported	Overall trend for patients treated with surgery to have larger disc herniation areas and smaller canal cross-section areas, corresponding to larger percent canal compromise than the non-operative group.	Three measurements of each herniated disc and canal were taken to ensure reproducibility. Intra-rater measurement variability was within 3%
Dora<br>2005 [15]	Retrospective case-control study on patients with and without recurrent LDH	60<br>(41+19)	18-63	MRI	LDH & lumbar intervertebral discs	Image Access software (Imagic Imaging Solutions, Glattbrugg, Switzerland).	Minor disk degeneration but not herniation volume represents a risk factor for the recurrence of disk herniation after discectomy.	Intraclass correlation coefficient<br><br>Inter-rater: 0.97 (95% CI 0.95-0.99)
Knirsch<br>2005 [16]	Descriptive study on children referred for MRI of the lumbosacral spine for routine clinical indications	75<br>(32+43)	6-17	MRI	Lumbar vertebral bodies & dural sacs	J-Vision, Tiani Co., Vienna, Austria	MRI is a reliable method demonstrating the natural shape of the lumbosacral spine and its absolute values. These normal values compare well with those established by conventional radiological techniques.	Not reported
Violas<br>2005 [17]	Methodological MRI-study on children with idiopathic scoliosis	14<br>(1+13)	7-13<br>Mean 11	MRI	Lumbar intervertebral discs	Matlab custom-made software	Reproducible geometrical properties of intervertebral discs	Method demonstrated to be reproducible.<br>Fisher's test
Visuri<br>2005 [18]	Cross-sectional descriptive study on conscripts with and without chronic low back pain	198<br>(198+0)	18-26	MRI	Lumbar intervertebral discs, LDH & spinal canals	Not reported	Narrowing of the vertebral canal in the antero-posterior direction was more likely to produce<br>CLBP and radiating pain than intervertebral disc degeneration or narrowing of the intervertebral nerve root canals.	Not reported
Autio<br>2006 [19]	Longitudinal descriptive study on patients with unilateral sciatica	Baseline:<br>160<br>1st Follow-up: 73<br>2nd Follow-up: 55	19-78	MRI	LDH	Advantage Windows; General Electric Medical Systems	MRI is a useful prognostic tool for identifying patients with HNP-induced sciatica with a benign natural course.	Intraclass correlation coefficients<br><br>Intra-rater: 0.93-0.98<br><br>Inter-rater: 0.46-0.90
Jeong	Cross-sectional descriptive	15	17-47	MRI	Lumbar spinal canals,	Piview Star, Star Pacs	For achondroplasia patients a significant difference between	Not reported

Author and year	Study design and study sample	Sample size (males + females)	Age range	MRI/CT	MRI/CT variables	Measurement software	Main findings	Reproducibility of method
2006 [20]	study on symptomatic and non-symptomatic patients with achondroplasia	(5+10)	Mean 29		spinal cord & pedicles	Infinitt, Seoul, Korea	the groups in the cross-sectional area of the body canal at the upper lumbar levels was found. Patients with a narrower canal are more likely to develop symptoms of spinal stenosis than others.	
Zaaroor 2006 [21]	Descriptive study on normal MRI images	42 (28+14)	16-80 Mean 38.1	MRI	Cervical & thoracic dural sacs & subarachnoid spaces	PACS stations (Agfa)	The findings show that an endoscope designed to travel within the subarachnoid space must be smaller than 2.5mm in diameter.	Not reported
Hirasawa 2007 [22]	Descriptive study on subjects with no symptoms of low back pain	32 (32+0)	21-61 Mean 32	MRI	Lumbar dural sacs	OSIRIS (Digital Imaging Unit University Hospital of Geneva, Switzerland)	A significant posture-dependent difference of the dural sac cross-sectional area at the level of intervertebral disc in asymptomatic volunteers was demonstrated.	Not reported
Violas 2007 [23]	Longitudinal descriptive study on adolescents with idiopathic scoliosis undergone posterior spinal fusion	28 (2+26)	11-19 Mean 15	MRI	Lumbar intervertebral discs	Matlab custom-made software	It tended to prove that the recovery of balanced physiological positioning and inherent biomechanical loads could induce a restored hydration of disc, which should favor the remodeling of free segments.	Demonstrated to be reproducible and reliable since there was no significant difference in volume (P = 0.1).
Lurie 2008 [24]	Reliability study on patients with LDH	58 (30+28)	Mean 42.3	MRI	Lumbar thecal sacs & LDH	ImageJ software (Rasband, W.S., ImageJ, U.S. National Institutes of Health, Bethesda, MD)	Classification of disc morphology showed substantial intra- and inter-reader agreement, whereas thecal sac and nerve root compression showed more moderate reader reliability. Quantitative measures of canal and thecal sac area showed good reliability, whereas measurement of disc fragment area showed more modest reliability.	Intraclass correlation coefficients. 0.87-0.96<br><br>Absolute measurement difference by 2 readers: 15 % of mean
Madsen 2008 [25]	Methodological comparative study on patients with lumbar spinal stenosis	36 (16+20)	18-80	MRI	Lumbar spinal canals & lumbar intervertebral discs	Osiris Software, free version 4.16	Horizontal MRI with the patient supine and the legs straightened was comparable to vertical MRI whether axial compression was added or not. Axial load was not considered to have a clinically relevant effect on spinal canal diameters.	The reliability of the applied measurements was tested in a separate third section preceding the main studies.
Masharawi 2008 [26]	Reproducibility study on children from the general population	40	12-16	MRI	Vertebral body, intervertebral disc, zygoappophyseal facets, pedicle, lumbar lordisos and sacral inclination	iQ-VIEW, (IMAGE Information Systems Ltd., version 1.2.2, Plauen, Germany)	Quantitative lumbar MRI measurements in children from the general population were found to be reproducible indicating a good visualization of immature vertebral anatomic margins on MRIs and an accurate definition of the measurement protocol.	Limits of agreement<br>&<br>Intraclass correlation coefficients<br><br>(Too many values to report)
Zou 2008 [27]	Cross-sectional descriptive study on patients with low back pain with and without radiculopathy	553 (234+319)	18-76 Mean 46.2	(Kinetic) MRI	LDH	MRI Analyzer Version 3 (Truemetric Corp., Bellflower, CA)	A significant increase in the degree of lumbar disc herniation was found by examining flexion and extension views when compared with neutral views alone.	Not reported
Ahn 2009 [28]	Descriptive study on patients with neurogenic intermittent claudication and/or sciatica	51 (24+27)	21-77 Mean 51	MRI	Lumbar dural sacs	PiView TM; INFINITT Co., Ltd., Seoul	Dural sac cross-sectional area decreased with increased severity of disc degeneration with axial loading, except for grade 5 disc degeneration.	Intra- and inter-observer reliability in change if dural sac cross-sectional area was investigated using the kappa test.<br><br>Intra-observer: k=0.84<br>Inter-observer: k=0.86, k=0.87
Grams 2010 [29]	Descriptive comparison-study on patients undergone MRI and CT of same spinal levels	17 (9+8)	35-78 Mean 63	MRI & CT	Lumbar spinal canals & LDH	iplan spine (Brainlab)	Spinal cord size and size of disc protrusions displayed no significant difference between MRI and CT	Not reported
Pneumaticos 2010 [30]	Descriptive study on patients with LDH symptoms compared with controls without LDH controls	60 (35+25)	26-68 Mean 42.9	MRI	LDH & dural sacs	Brain-image software (National Institute of Health Behavioural Neurogenetics and Neuroimaging Research Centre)	Quantitative measurements of MRI can improve significantly the ability to identify the patients who would benefit from discectomy.	Not reported
Schizas 2010 [31]	Retrospective study on prospective patient cohorts. Surgical and conservative treated lumbar spinal	95 (36+59)	Mean 64.8	MRI	Lumbar dural sacs	OsiriX Imaging Software	The grading defines stenosis in different subjects than surface measurements alone. Since it mainly considers impingement of neural tissue it might be a more appropriate clinical and research tool as well as carrying a prognostic	Stenosis morphologic grading: average inter- and intra-observer kappas were 0.44 (SD, 0.17) and 0.65 (SD, 0.14)

Author and year	Study design and study sample	Sample size (males + females)	Age range	MRI/CT	MRI/CT variables	Measurement software	Main findings	Reproducibility of method
	stenosis patients						value.	
Lee<br>2011 [32]	Methodological study on patients with lumbar central canal stenosis	Pre-step: 11<br>First-step: 19<br>Second-step: 61	54-72<br>52-84<br>32-79	MRI	Lumbar dural sacs	Workstation (PiView, Infinitt Co. Ltd., Seoul, Korea)	This new grading system may be helpful to clinicians as a simple and practical evaluation of lumbar central canal stenosis and for communicating with each other.	Not reported
Ogura<br>2011 [33]	Descriptive comparison-study on patients diagnosed with lumbar spinal canal stenosis, spondylosis or LDH	61<br>(47+14)	46-82<br>Mean 63.5	MRI & CT	Lumbar dural sacs & ligamentum flavum	CIS-Image/Viewer for Windows Version 2.6.07; IBM Japan Ltd., Tokyo, Japan	Both CT and MRI provided reproducible measurements of lumbar intra-canal dimensions. Ligamentum flavum thickness may be more accurately measured by CT.	Intraclass correlation coefficient<br><br>Intra- and inter-rater for dural sac: (>0.9)<br>Intra- and inter-rater for ligamentum flavum: 0.867-0.913
Puigdellivol-Sanchez<br>2011 [34]	Methodological study on patients with low back pain.	7<br>(3+4)	24-58<br>Mean 41.6	MRI	Lumbar spinal canals	Amira Software	Quick semi-automatic hospital 3D reconstructions give results close to detailed neuroanatomical 3D reconstruction and could be used in the future for individual quantification of lumbosacral cerebrospinal fluid volumes and other structures for anaesthetic purposes.	Not reported

References

1. Grenier N, Kressel HY, Schiebler ML, Grossman RI, Dalinka MK: **Normal and degenerative posterior spinal structures: MR imaging.** *Radiology* 1987, **165:**517-525.
2. Hamanishi C, Matukura N, Fujita M, Tomihara M, Tanaka S: **Cross-sectional area of the stenotic lumbar dural tube measured from the transverse views of magnetic resonance imaging.** *J Spinal Disord* 1994, **7:**388-393.
3. Carragee EJ, Kim DH: **A prospective analysis of magnetic resonance imaging findings in patients with sciatica and lumbar disc herniation. Correlation of outcomes with disc fragment and canal morphology.** *Spine (Phila Pa 1976)* 1997, **22:**1650-1660.

4. Malko JA, Hutton WC, Fajman WA: **An in vivo magnetic resonance imaging study of changes in the volume (and fluid content) of the lumbar intervertebral discs during a simulated diurnal load cycle.** *Spine (Phila Pa 1976)* 1999, **24:**1015-1022.
5. Chung SS, Lee CS, Kim SH, Chung MW, Ahn JM: **Effect of low back posture on the morphology of the spinal canal.** *Skeletal radiology* 2000, **29:**217-223.
6. Holodny AI, Kisza PS, Contractor S, Liu WC: **Does a herniated nucleus pulposus contribute significantly to a decrease in height of the intervertebral disc? Quantitative volumetric MRI.** *Neuroradiology* 2000, **42:**451-454.
7. Pneumaticos SG, Hipp JA, Esses SI: **Sensitivity and specificity of dural sac and herniated disc dimensions in patients with low back-related leg pain.** *Journal of magnetic resonance imaging : JMRI* 2000, **12:**439-443.
8. Cooley JR, Danielson CD, Schultz GD, Hall TA: **Posterior disk displacement: morphologic assessment and measurement reliability-lumbar spine.** *Journal of manipulative and physiological therapeutics* 2001, **24:**317-326.
9. Dora C, Walchli B, Elfering A, Gal I, Weishaupt D, Boos N: **The significance of spinal canal dimensions in discriminating symptomatic from asymptomatic disc herniations.** *European spine journal : official publication of the European Spine Society, the European Spinal Deformity Society, and the European Section of the Cervical Spine Research Society* 2002, **11:**575-581.
10. Haig AJ, Weiner JB, Tew J, Quint D, Yamakawa K: **The relation among spinal geometry on MRI, paraspinal electromyographic abnormalities, and age in persons referred for electrodiagnostic testing of low back symptoms.** *Spine (Phila Pa 1976)* 2002, **27:**1918-1925; discussion 1924-1915.
11. Speciale AC, Pietrobon R, Urban CW, Richardson WJ, Helms CA, Major N, Enterline D, Hey L, Haglund M, Turner DA: **Observer variability in assessing lumbar spinal stenosis severity on magnetic resonance imaging and its relation to cross-sectional spinal canal area.** *Spine (Phila Pa 1976)* 2002, **27:**1082-1086.
12. Malko JA, Hutton WC, Fajman WA: **An in vivo MRI study of the changes in volume (and fluid content) of the lumbar intervertebral disc after overnight bed rest and during an 8-hour walking protocol.** *Journal of spinal disorders & techniques* 2002, **15:**157-163.
13. Jeffrey JE, Campbell DM, Golden MH, Smith FW, Porter RW: **Antenatal factors in the development of the lumbar vertebral canal: a magnetic resonance imaging study.** *Spine (Phila Pa 1976)* 2003, **28:**1418-1423.
14. Carlisle E, Luna M, Tsou PM, Wang JC: **Percent spinal canal compromise on MRI utilized for predicting the need for surgical treatment in single-level lumbar intervertebral disc herniation.** *The spine journal : official journal of the North American Spine Society* 2005, **5:**608-614.
15. Dora C, Schmid MR, Elfering A, Zanetti M, Hodler J, Boos N: **Lumbar disk herniation: do MR imaging findings predict recurrence after surgical diskectomy?** *Radiology* 2005, **235:**562-567.

16. Knirsch W, Kurtz C, Haffner N, Langer M, Kececioglu D: **Normal values of the sagittal diameter of the lumbar spine (vertebral body and dural sac) in children measured by MRI.** *Pediatric radiology* 2005, **35:**419-424.
17. Violas P, Estivalèzes E, Pédrono A, Sales De Gauzy J, Sévely A, Swider P: **A method to investigate intervertebral disc morphology from MRI in early idiopathic scoliosis: A preliminary evaluation in a group of 14 patients.** *Magn Reson Imaging* 2005, **23:**475-479.
18. Visuri T, Ulaska J, Eskelin M, Pulkkinen P: **Narrowing of lumbar spinal canal predicts chronic low back pain more accurately than intervertebral disc degeneration: a magnetic resonance imaging study in young Finnish male conscripts.** *Military medicine* 2005, **170:**926-930.
19. Autio RA, Karppinen J, Niinimäki J, Ojala R, Kurunlahti M, Haapea M, Vanharanta H, Tervonen O: **Determinants of spontaneous resorption of intervertebral disc herniations.** *Spine* 2006, **31:**1247-1252.
20. Jeong ST, Song HR, Keny SM, Telang SS, Suh SW, Hong SJ: **MRI study of the lumbar spine in achondroplasia. A morphometric analysis for the evaluation of stenosis of the canal.** *The Journal of bone and joint surgery British volume* 2006, **88:**1192-1196.
21. Zaaroor M, Kosa G, Peri-Eran A, Maharil I, Shoham M, Goldsher D: **Morphological study of the spinal canal content for subarachnoid endoscopy.** *Minimally invasive neurosurgery : MIN* 2006, **49:**220-226.
22. Hirasawa Y, Bashir WA, Smith FW, Magnusson ML, Pope MH, Takahashi K: **Postural changes of the dural sac in the lumbar spines of asymptomatic individuals using positional stand-up magnetic resonance imaging.** *Spine (Phila Pa 1976)* 2007, **32:**E136-140.
23. Violas P, Estivalezes E, Briot J, Sales de Gauzy J, Swider P: **Objective quantification of intervertebral disc volume properties using MRI in idiopathic scoliosis surgery.** *Magn Reson Imaging* 2007, **25:**386-391.
24. Lurie JD, Tosteson AN, Tosteson TD, Carragee E, Carrino JA, Kaiser J, Sequeiros RT, Lecomte AR, Grove MR, Blood EA, et al: **Reliability of magnetic resonance imaging readings for lumbar disc herniation in the Spine Patient Outcomes Research Trial (SPORT).** *Spine (Phila Pa 1976)* 2008, **33:**991-998.
25. Madsen R, Jensen TS, Pope M, Sorensen JS, Bendix T: **The effect of body position and axial load on spinal canal morphology: an MRI study of central spinal stenosis.** *Spine (Phila Pa 1976)* 2008, **33:**61-67.
26. Masharawi Y, Kjaer P, Bendix T, Manniche C, Wedderkopp N, Sorensen JS, Peled N, Jensen TS: **The reproducibility of quantitative measurements in lumbar magnetic resonance imaging of children from the general population.** *Spine (Phila Pa 1976)* 2008, **33:**2094-2100.
27. Zou J, Yang H, Miyazaki M, Wei F, Hong SW, Yoon SH, Morishita Y, Wang JC: **Missed lumbar disc herniations diagnosed with kinetic magnetic resonance imaging.** *Spine (Phila Pa 1976)* 2008, **33:**E140-144.
28. Ahn TJ, Lee SH, Choi G, Ahn Y, Liu WC, Kim HJ, Lee HY: **Effect of intervertebral disk degeneration on spinal stenosis during magnetic resonance imaging with axial loading.** *Neurologia medico-chirurgica* 2009, **49:**242-247; discussion 247.

29. Grams AE, Gempt J, Forschler A: **Comparison of spinal anatomy between 3-Tesla MRI and CT-myelography under healthy and pathological conditions.** *Surgical and radiologic anatomy : SRA* 2010, **32:**581-585.
30. Pneumaticos SG, Chatziioannou AN, Hipp J, Chatziioannou SN: **Prediction of successful discectomy using MRI quantitation of dural sac and herniated disc dimensions.** *International journal of clinical practice* 2010, **64:**13-18.
31. Schizas C, Theumann N, Burn A, Tansey R, Wardlaw D, Smith FW, Kulik G: **Qualitative grading of severity of lumbar spinal stenosis based on the morphology of the dural sac on magnetic resonance images.** *Spine (Phila Pa 1976)* 2010, **35:**1919-1924.
32. Lee GY, Lee JW, Choi HS, Oh KJ, Kang HS: **A new grading system of lumbar central canal stenosis on MRI: an easy and reliable method.** *Skeletal radiology* 2011, **40:**1033-1039.
33. Ogura H, Miyamoto K, Fukuta S, Naganawa T, Shimizu K: **Comparison of magnetic resonance imaging and computed tomography-myelography for quantitative evaluation of lumbar intracanalar cross-section.** *Yonsei medical journal* 2011, **52:**137-144.
34. Puigdellivol-Sanchez A, Prats-Galino A, Reina MA, Maches F, Hernandez JM, De Andres J, van Zundert A: **Three-dimensional magnetic resonance image of structures enclosed in the spinal canal relevant to anesthetists and estimation of the lumbosacral CSF volume.** *Acta anaesthesiologica Belgica* 2011, **62:**37-45.
