# Supplementary material for: A method for quantitative measurement of lumbar intervertebral disc structures: an intra- and inter-rater agreement and reliability study
Source: Chiropr Man Therap. 2013 Aug 16;21:26. doi: 10.1186/2045-709X-21-26 (PMC3751877; doi:10.1186/2045-709X-21-26)
Supplement: Additional file 2 — Description of calculating software (computer program available from the authors on request). [file 2045-709X-21-26-S2.pdf]

Additional file 4 - Description of calculating software

A customized MatLab algorithm (Mathworks, Inc.) was developed to read and analyze the output files from the Osirix measurement software. The output files were converted from CSV into XLSX before the algorithm was applied.

The length measurements from the included sagittal images from all the structures were calculated based on the Euclidean distance between two points. The points were manually selected for each slice.

Given two points A(x1,y1,z1) and B(x2,y2,z2) for each slice, the length between these two points is given by:

$$dist = \sqrt{(x2 - x1)^2 + (y2 - y1)^2 + (z2 - z1)^2}$$

The length measurements from all included sagittal images from every structure were used to calculate the cross-sectional areas (CSA) of a structure. Using the length of two consecutive slice lengths, the CSA can be calculated using the following formula:

$$CSA = \sum_{i=slice\ number} \left(\frac{1}{2} \times (slicelength_i + slicelength_{i+1}) \times h\right)$$

where h is the width of the segment (slice thickness 4mm + interslice gap 0.8 mm = 4.8mm.)
