# Supplementary material for: A method for quantitative measurement of lumbar intervertebral disc structures: an intra- and inter-rater agreement and reliability study
Source: Chiropr Man Therap. 2013 Aug 16;21:26. doi: 10.1186/2045-709X-21-26 (PMC3751877; doi:10.1186/2045-709X-21-26)
Supplement: Additional file 3 — Cross tabulations for start- and end-slices. [file 2045-709X-21-26-S3.pdf]

Additional file 3 - Cross tabulations for start- and end-slices

START-SLICE INTRA-RATER AIVH, PIVH, IVDL, ADML, PDML, VADM, VPDM					
**Start2**	**1**	**2**	**3**	**4**	**Total**
**start1**					
**1**	3	0	0	0	3
**2**	0	15	1	0	16
**3**	0	1	7	0	8
**4**	0	1	1	3	5
**Total**	3	17	9	3	32

END-SLICE INTRA-RATER AIVH, PIVH, IVDL, ADML, PDML, VADM, VPDM					
**End2**	**8**	**9**	**10**	**11**	**Total**
**End1**					
**8**	1	0	0	0	1
**9**	0	8	2	0	10
**10**	0	2	18	1	21
**Total**	1	10	20	1	32

START-SLICE INTRA-RATER ADSL						
**Start2**	**1**	**2**	**3**	**4**	**5**	**Total**
**Start1**						
**1**	1	0	0	0	0	1
**2**	0	11	1	0	0	12
**3**	0	0	9	1	0	10
**4**	0	1	1	6	0	8
**5**	0	0	0	0	1	1
**Total**	1	12	11	7	1	32

END-SLICE INTRA-RATER ADSL						
**End2**	**7**	**8**	**9**	**10**	**11**	**Total**
**End1**						
**7**	2	0	0	0	0	2
**8**	0	4	1	0	0	5
**9**	0	0	10	1	0	11
**10**	0	0	1	12	1	14
**Total**	2	4	12	13	1	32

START-SLICE INTER-RATER AIVH, PIVH, IVDL, ADML, PDML, VADM, VPDM				
**Start2**	**1**	**2**	**3**	**Total**
**Start1**				
**1**	1	2	0	3
**2**	2	14	0	16
**3**	0	5	3	8
**4**	0	2	3	5
**Total**	3	23	6	32

END-SLICE INTER-RATER AIVH, PIVH, IVDL, ADML, PDML, VADM, VPDM					
**End2**	**8**	**9**	**10**	**11**	**Total**
**End1**					
**8**	1	0	0	0	1
**9**	0	4	6	0	10
**10**	0	2	15	4	21
**Total**	1	6	21	4	32

START-SLICE INTER-RATER ADSL					
**Start2**	**1**	**2**	**3**	**4**	**Total**
**Start1**					
**1**	1	0	0	0	1
**2**	2	10	0	0	12
**3**	0	6	3	1	10
**4**	0	5	3	0	8
**5**	0	1	0	0	1
**Total**	3	22	6	1	32

END-SLICE INTER-RATER ADSL					
**End2**	**8**	**9**	**10**	**11**	**Total**
**End1**					
**7**	1	0	1	0	2
**8**	0	0	5	0	5
**9**	0	6	5	0	11
**10**	0	1	9	4	14
**Total**	1	7	20	4	32
