# Supplementary material for: A method for quantitative measurement of lumbar intervertebral disc structures: an intra- and inter-rater agreement and reliability study
Source: Chiropr Man Therap. 2013 Aug 16;21:26. doi: 10.1186/2045-709X-21-26 (PMC3751877; doi:10.1186/2045-709X-21-26)
Supplement: Additional file 4 — Graphs of limits of agreement. [file 2045-709X-21-26-S4.pdf]

Additional file 2 - Graphs of limits of agreement

Intra-rater

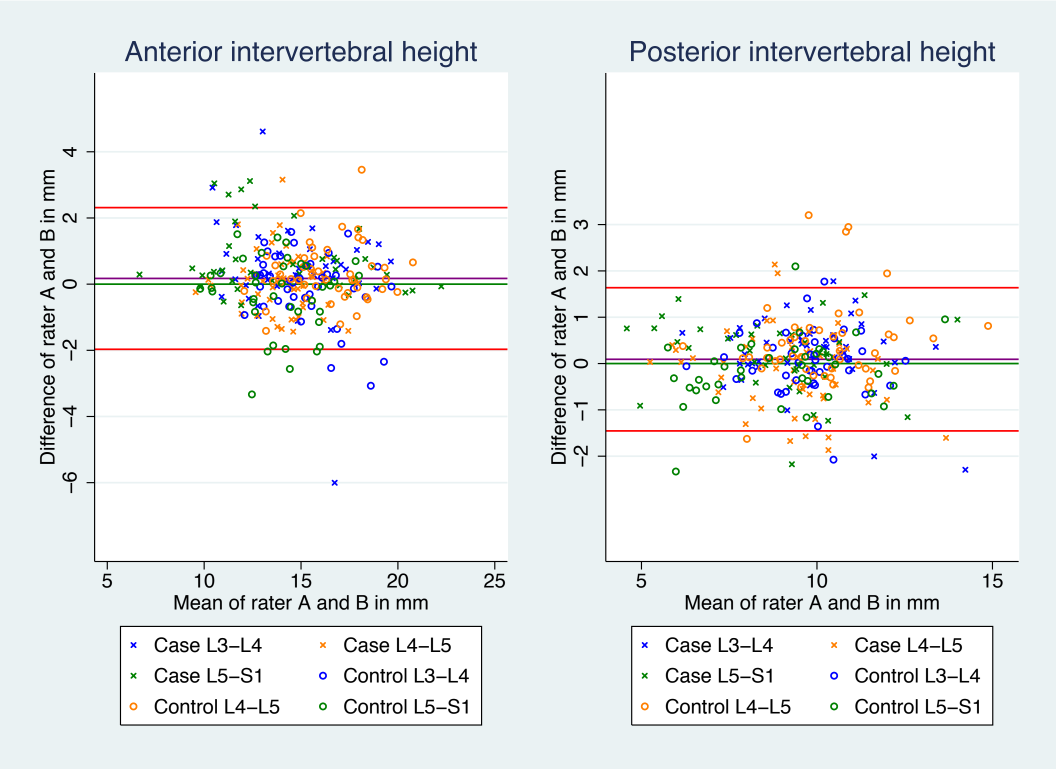

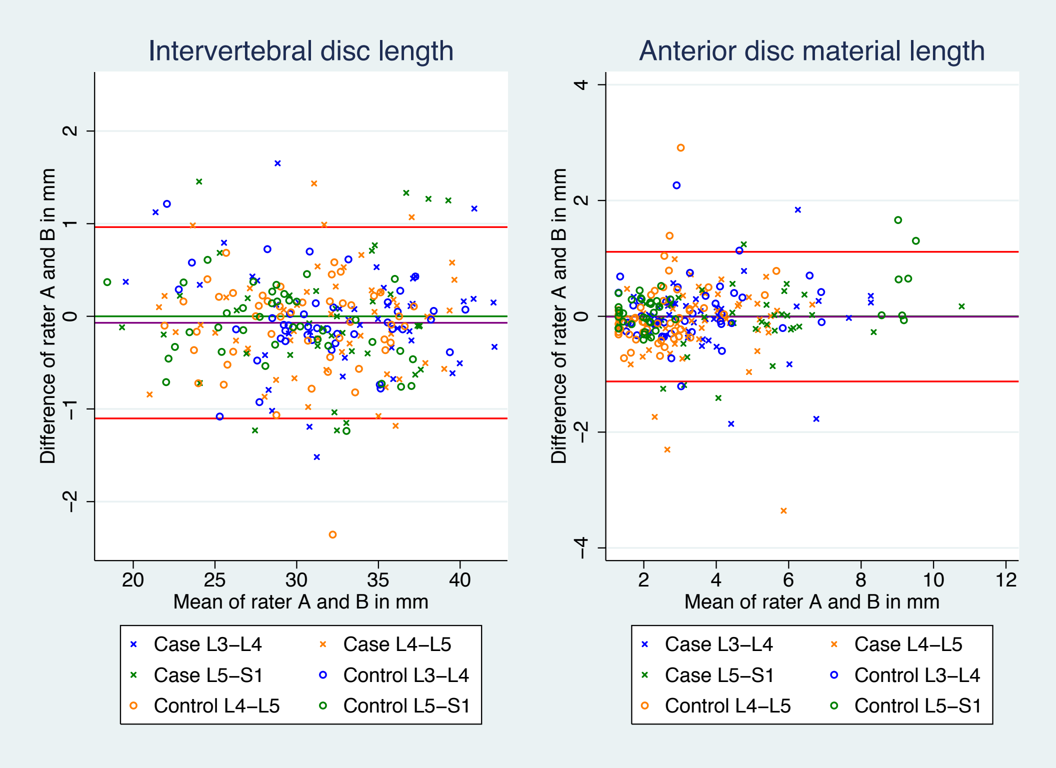

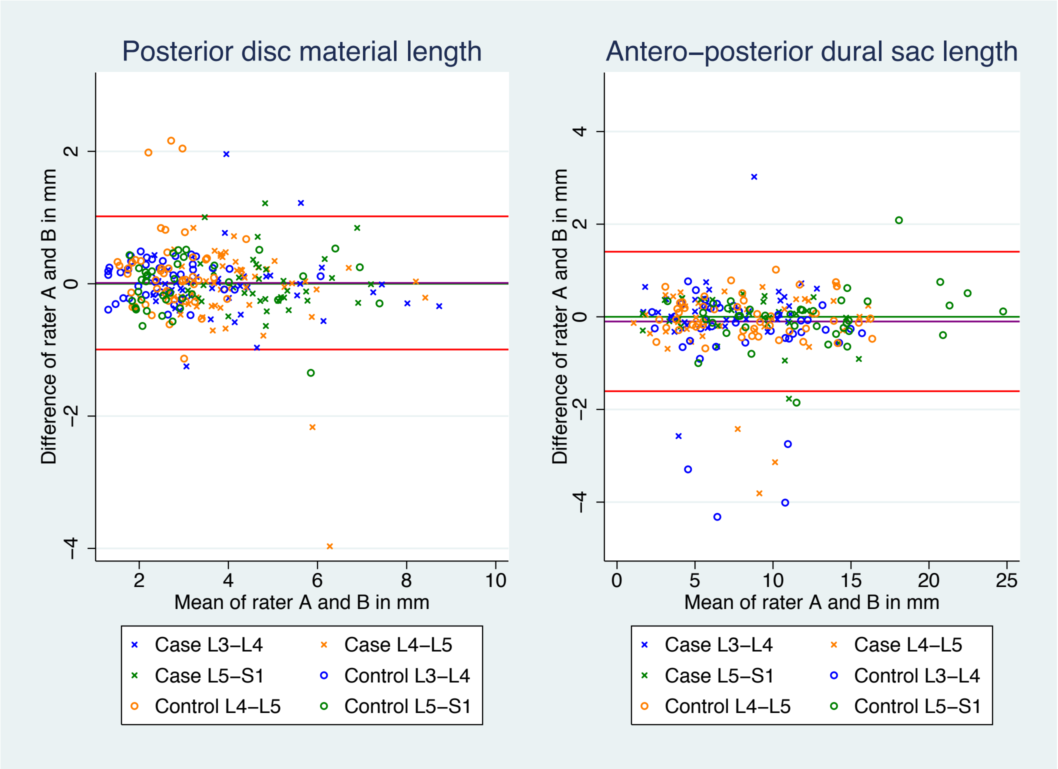
Posterior disc material length
Difference of rater A and B in mm
Mean of rater A and B in mm
Antero−posterior dural sac length
Difference of rater A and B in mm
Mean of rater A and B in mm
Case L3−L4
Case L4−L5
Case L5−S1
Control L3−L4
Control L4−L5
Control L5−S1

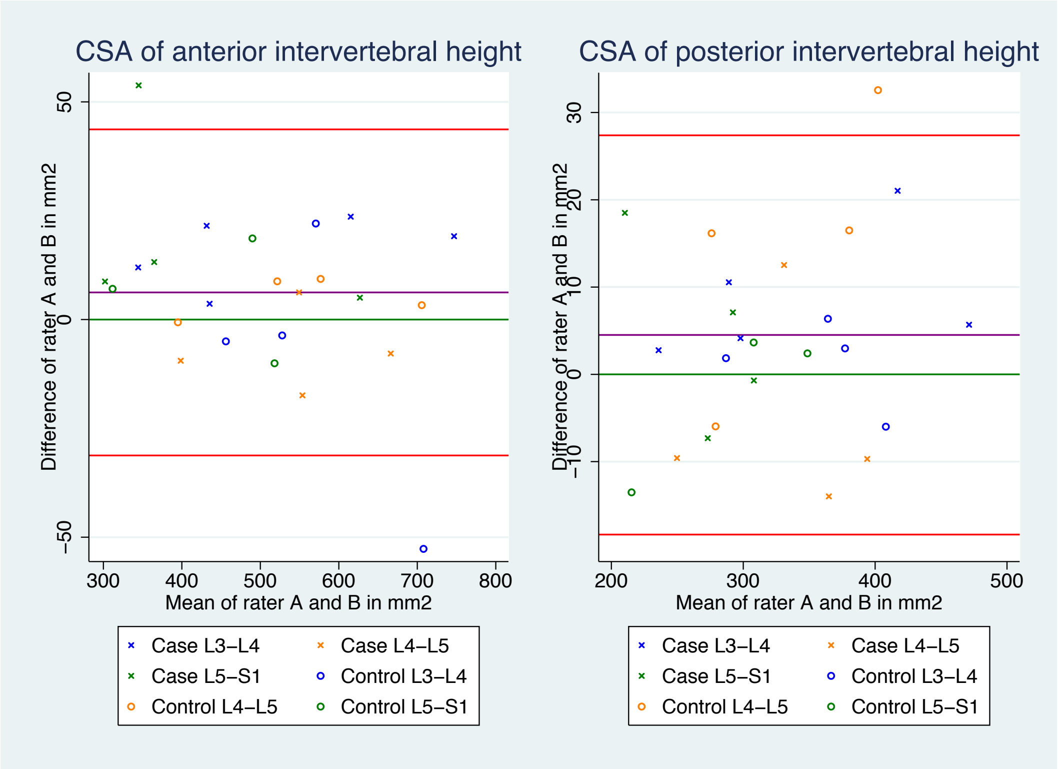
CSA of anterior intervertebral height
Difference of rater A and B in mm2
Mean of rater A and B in mm2
CSA of posterior intervertebral height
Difference of rater A and B in mm2
Mean of rater A and B in mm2
Case L3−L4
Case L4−L5
Case L5−S1
Control L3−L4
Control L4−L5
Control L5−S1

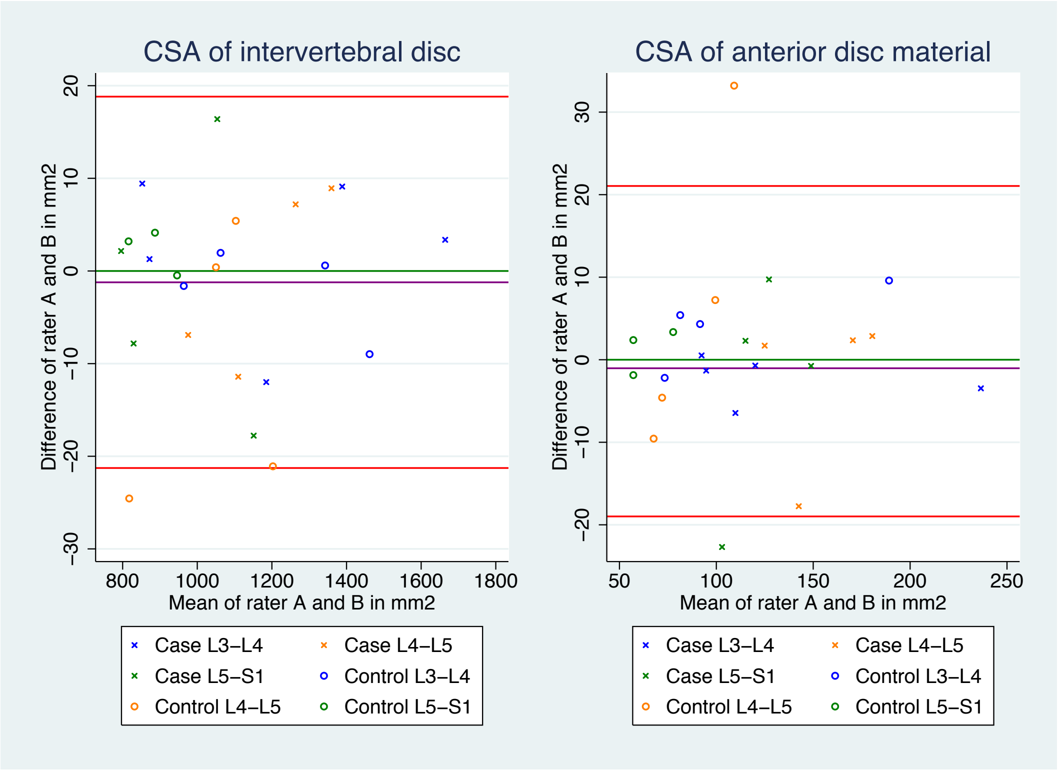
CSA of intervertebral disc
CSA of anterior disc material
Difference of rater A and B in mm2
Mean of rater A and B in mm2
Case L3–L4
Case L4–L5
Case L5–S1
Control L3–L4
Control L4–L5
Control L5–S1

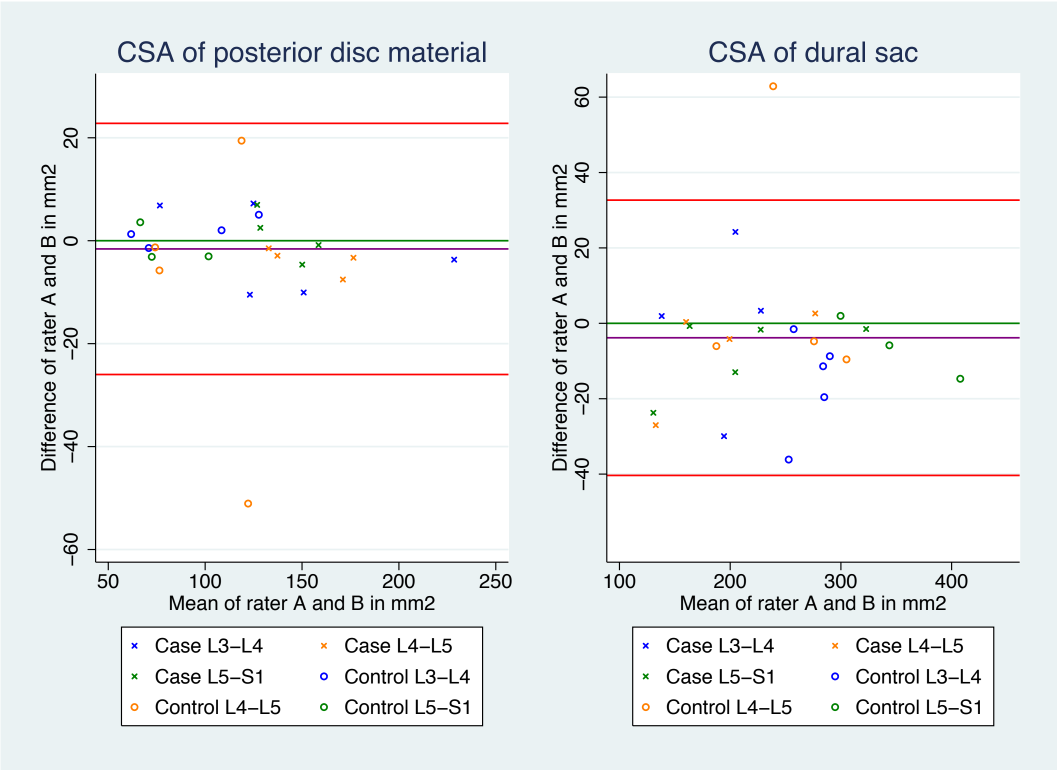
CSA of posterior disc material
CSA of dural sac
Difference of rater A and B in mm2
Mean of rater A and B in mm2
Case L3–L4
Case L4–L5
Case L5–S1
Control L3–L4
Control L4–L5
Control L5–S1

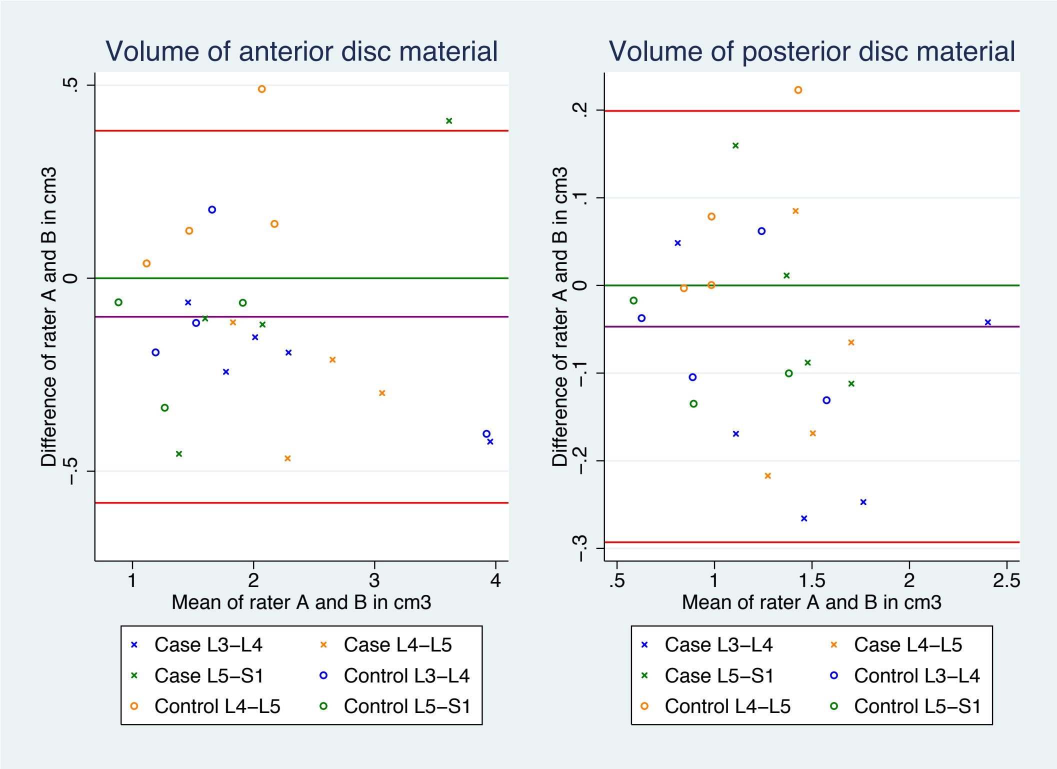
Volume of anterior disc material
Difference of rater A and B in cm3
Mean of rater A and B in cm3
Volume of posterior disc material
Difference of rater A and B in cm3
Mean of rater A and B in cm3
Case L3–L4
Case L4–L5
Case L5–S1
Control L3–L4
Control L4–L5
Control L5–S1

Inter-rater

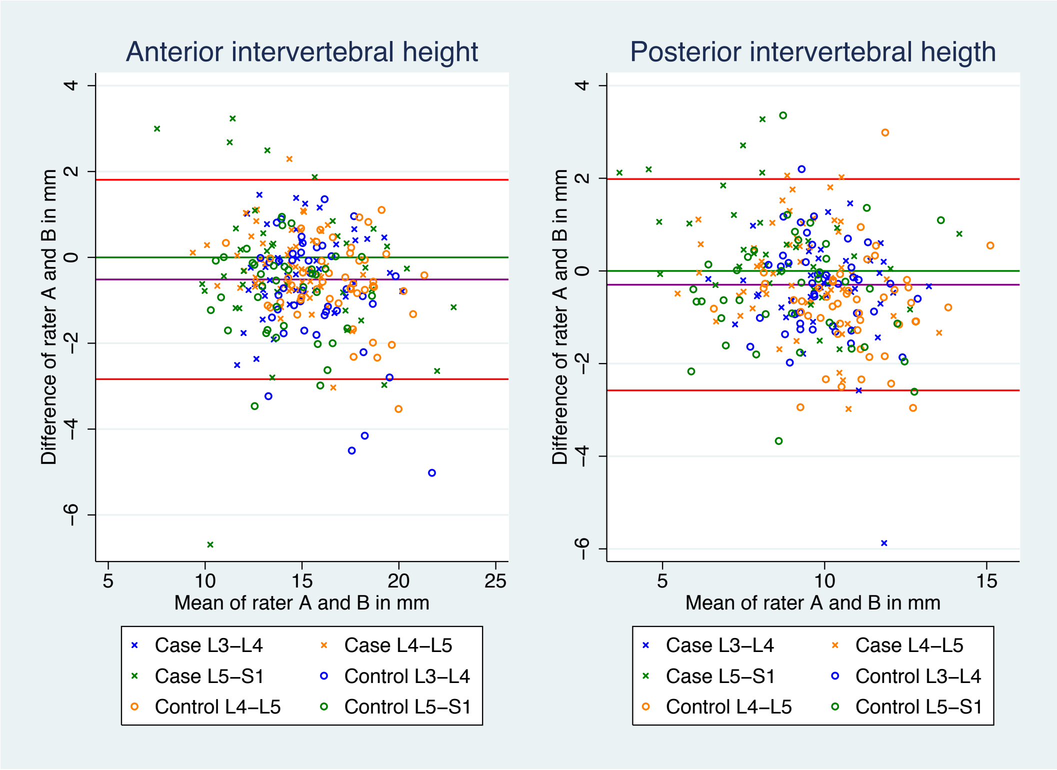
Anterior intervertebral height
Difference of rater A and B in mm
Mean of rater A and B in mm
Posterior intervertebral heigth
Difference of rater A and B in mm
Mean of rater A and B in mm
Case L3–L4
Case L4–L5
Case L5–S1
Control L3–L4
Control L4–L5
Control L5–S1

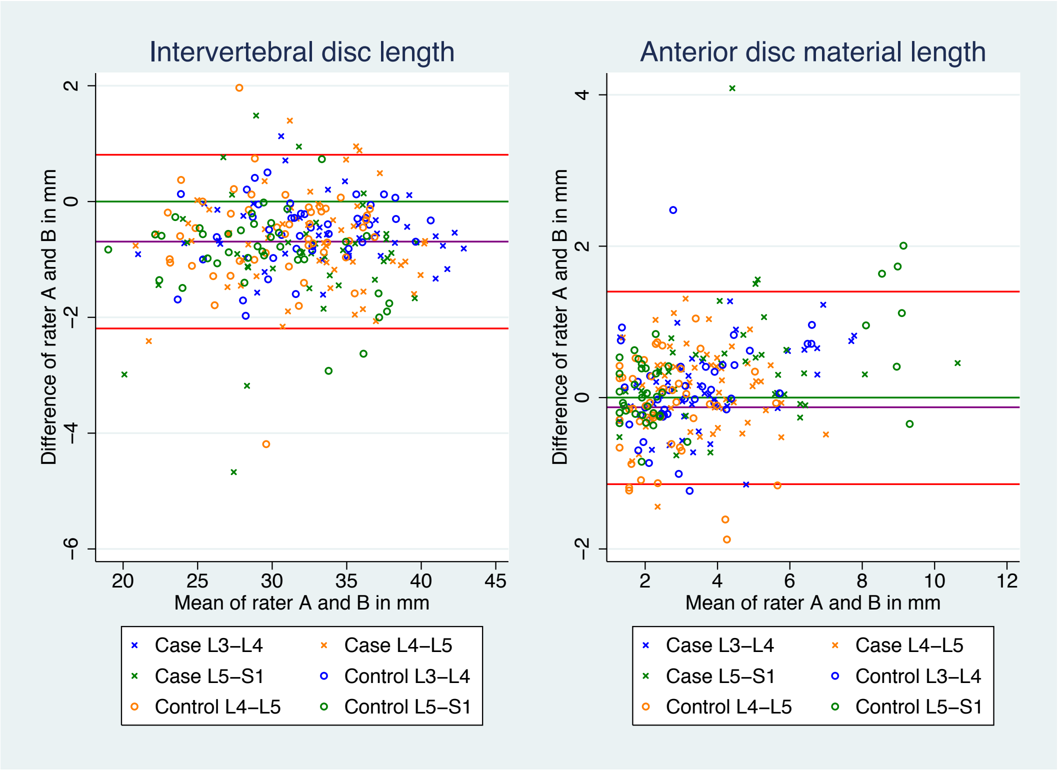
Intervertebral disc length
Anterior disc material length
Difference of rater A and B in mm
Mean of rater A and B in mm
Case L3–L4
Case L4–L5
Case L5–S1
Control L3–L4
Control L4–L5
Control L5–S1

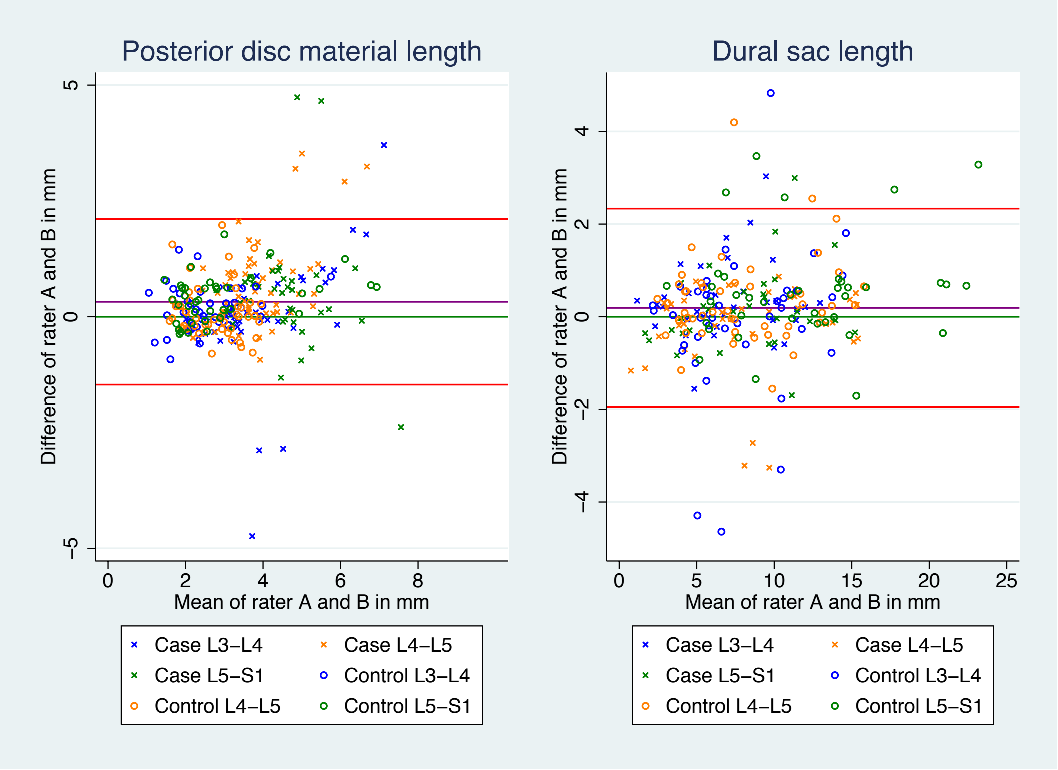
Posterior disc material length
Dural sac length
Difference of rater A and B in mm
Mean of rater A and B in mm
Case L3–L4
Case L4–L5
Case L5–S1
Control L3–L4
Control L4–L5
Control L5–S1

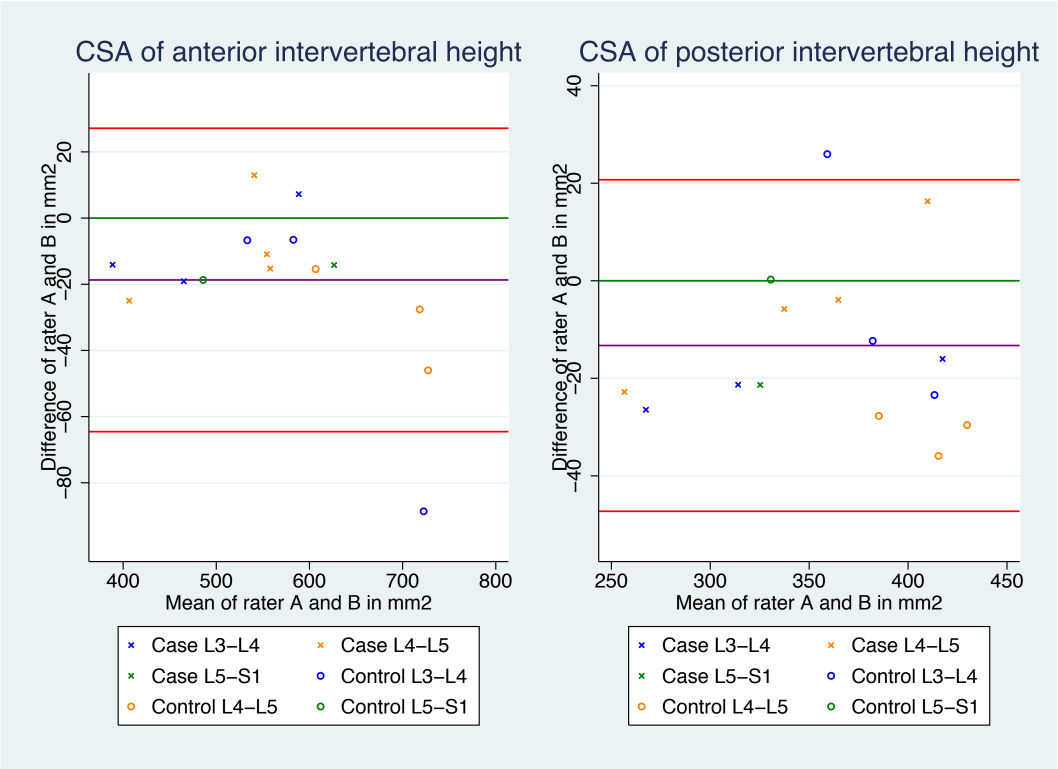
CSA of anterior intervertebral height
Difference of rater A and B in mm2
20
0
−20
−40
−60
−80
400
500
600
700
800
Mean of rater A and B in mm2
Case L3−L4
Case L4−L5
Case L5−S1
Control L3−L4
Control L4−L5
Control L5−S1
CSA of posterior intervertebral height
Difference of rater A and B in mm2
40
20
0
−20
−40
250
300
350
400
450
Mean of rater A and B in mm2
Case L3−L4
Case L4−L5
Case L5−S1
Control L3−L4
Control L4−L5
Control L5−S1

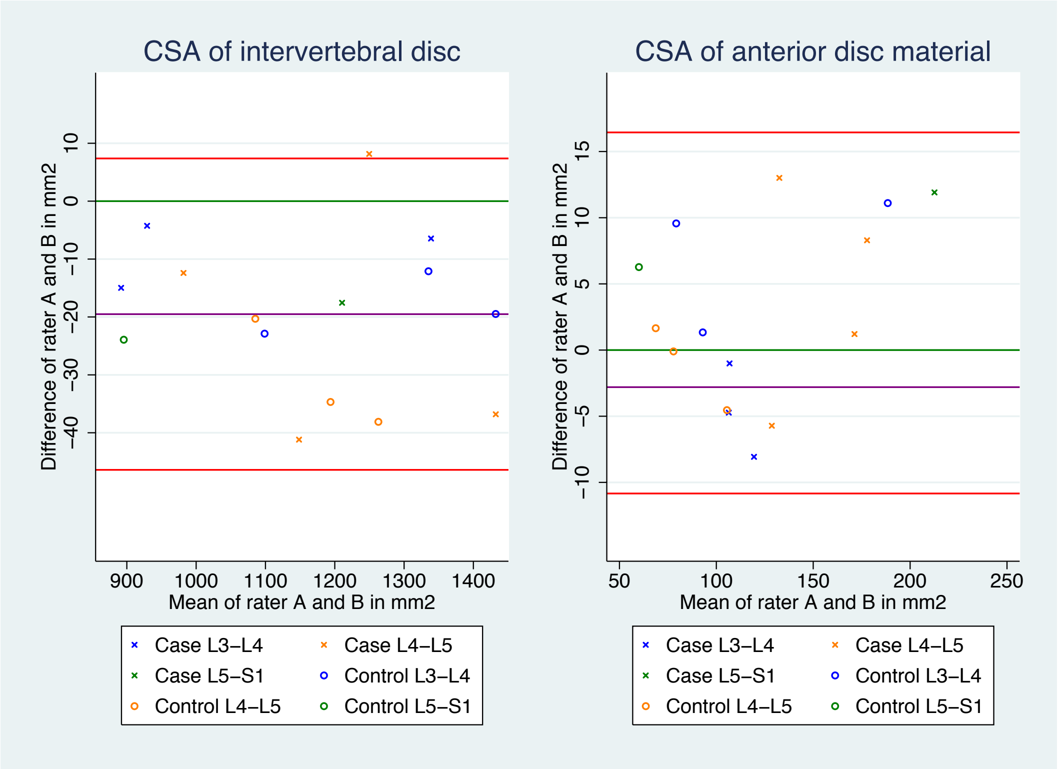
CSA of intervertebral disc
Difference of rater A and B in mm2
10
0
−10
−20
−30
−40
900
1000
1100
1200
1300
1400
Mean of rater A and B in mm2
Case L3−L4
Case L4−L5
Case L5−S1
Control L3−L4
Control L4−L5
Control L5−S1
CSA of anterior disc material
Difference of rater A and B in mm2
15
10
5
0
−5
−10
50
100
150
200
250
Mean of rater A and B in mm2
Case L3−L4
Case L4−L5
Case L5−S1
Control L3−L4
Control L4−L5
Control L5−S1

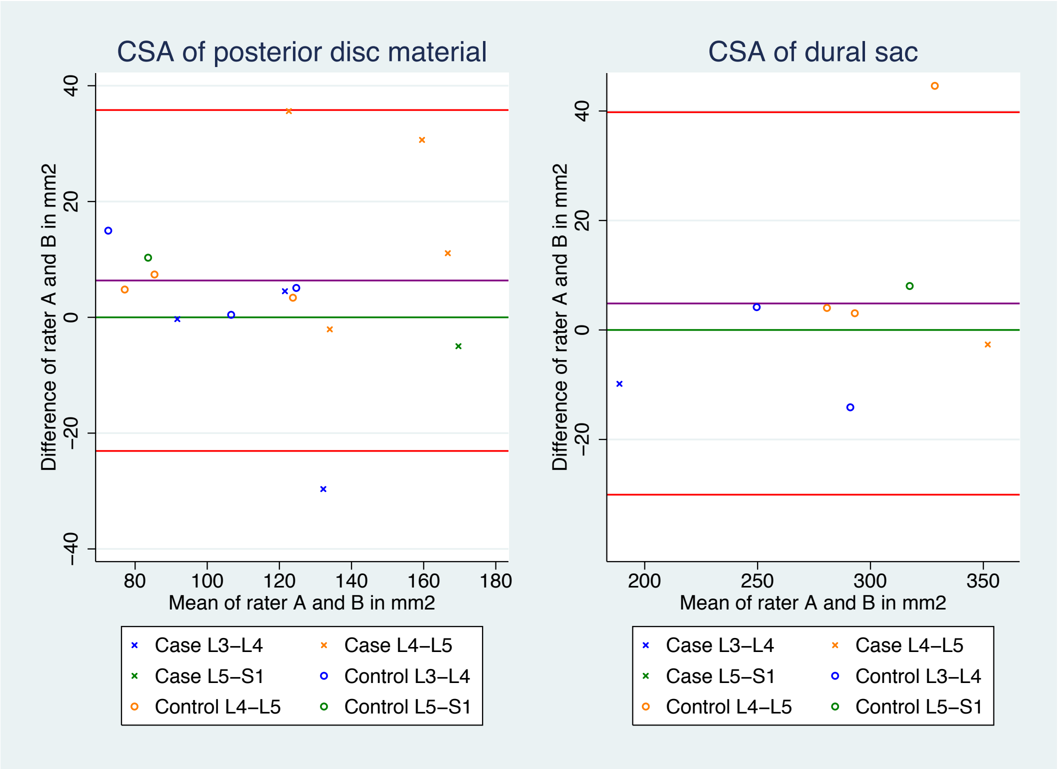
CSA of posterior disc material
Difference of rater A and B in mm2
Mean of rater A and B in mm2
−40
−20
0
20
40
80
100
120
140
160
180
Case L3–L4
Case L4–L5
Case L5–S1
Control L3–L4
Control L4–L5
Control L5–S1
CSA of dural sac
Difference of rater A and B in mm2
Mean of rater A and B in mm2
−20
0
20
40
200
250
300
350
Case L3–L4
Case L4–L5
Case L5–S1
Control L3–L4
Control L4–L5
Control L5–S1

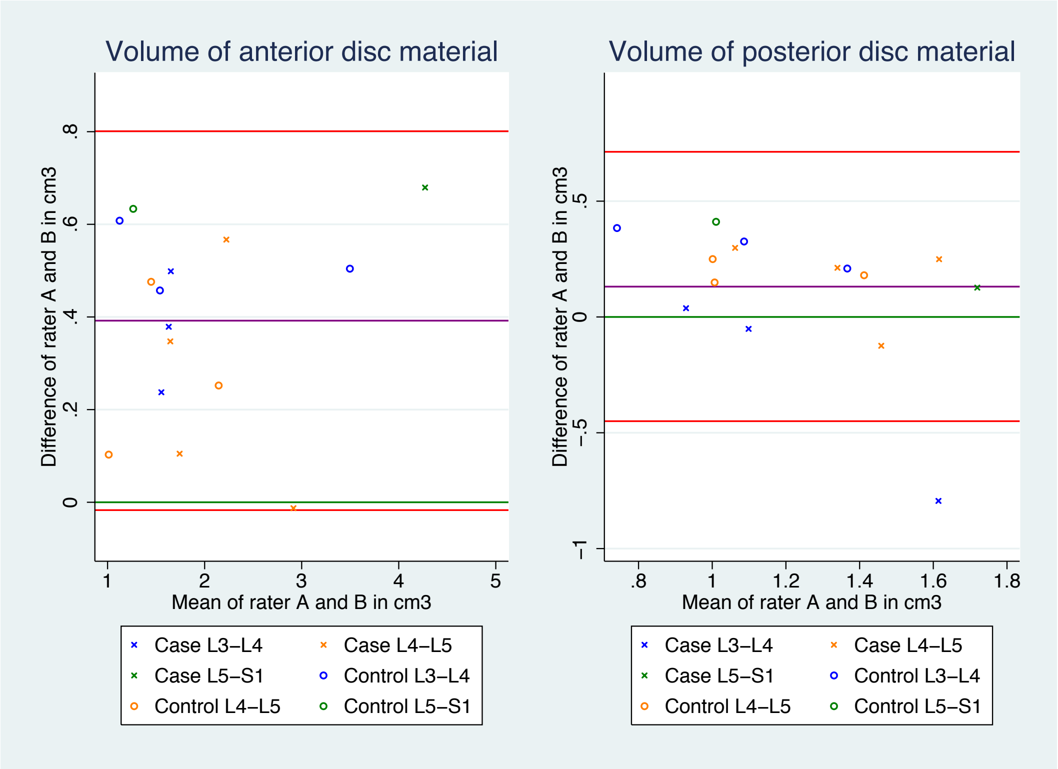
Volume of anterior disc material
Difference of rater A and B in cm3
Mean of rater A and B in cm3
0
.2
.4
.6
.8
1
2
3
4
5
Case L3–L4
Case L4–L5
Case L5–S1
Control L3–L4
Control L4–L5
Control L5–S1
Volume of posterior disc material
Difference of rater A and B in cm3
Mean of rater A and B in cm3
−1
−.5
0
.5
.8
1
1.2
1.4
1.6
1.8
Case L3–L4
Case L4–L5
Case L5–S1
Control L3–L4
Control L4–L5
Control L5–S1
